# Structure and Spectroscopy of Iron Pentacarbonyl, Fe(CO)_5_

**DOI:** 10.1021/jacs.2c01469

**Published:** 2022-09-16

**Authors:** A. Dominic Fortes, Stewart F. Parker

**Affiliations:** ISIS Neutron and Muon Facility, STFC Rutherford Appleton Laboratory, Harwell Science and Innovation Campus, Chilton, Oxfordshire OX11 0QX, U.K.

## Abstract

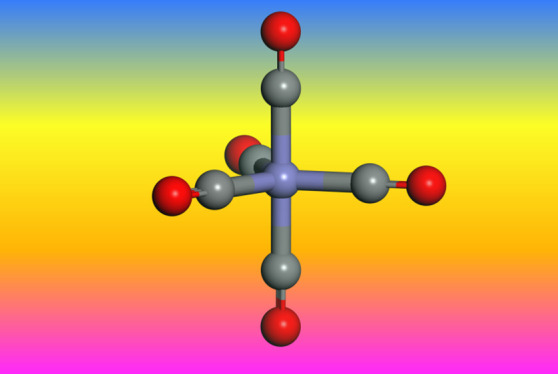

We have re-investigated the structure and vibrational spectroscopy of the iconic molecule iron pentacarbonyl, Fe(CO)_5_, in the solid state by neutron scattering methods. In addition to the known *C*2/*c* structure, we find that Fe(CO)_5_ undergoes a displacive ferroelastic phase transition at 105 K to a *P*1̅ structure. We propose that this is a result of certain intermolecular contacts becoming shorter than the sum of the van der Waals radii, resulting in an increased contribution of electrostatic repulsion to these interactions; this is manifested as a strain that breaks the symmetry of the crystal. Evaluation of the strain in a triclinic crystal required a description of the spontaneous strain in terms of a second-rank tensor, something that is feasible with high-precision powder diffraction data but practically very difficult using strain gauges on a single crystal of such low symmetry. The use of neutron vibrational spectroscopy (which is not subject to selection rules) has allowed the observation of all the fundamentals below 700 cm^–1^ for the first time. This has resulted in the re-assignment of several of the modes. Surprisingly, density functional theory calculations that were carried out to support the spectral assignments provided a poor description of the spectra.

## Introduction

Iron pentacarbonyl, Fe(CO)_5_, is one of the iconic molecules of inorganic chemistry. It was first reported in 1891^1^ and was only the second metal carbonyl to be discovered. While the stoichiometry was determined in the original report,^1^ the structure was vigorously debated for many years as to whether it was a trigonal bipyramid, *D*_3h_, or a square-based pyramid, *C*_4v_.^2^ The debate was apparently resolved in 1939 by a gas-phase electron diffraction (GED) structural determination^2^ that favored the *D*_3h_ structure. However, the *C*_4v_ structure was still being proposed as late as 1958.^3^ Only after the crystal structure was reported^4^ was the debate concluded. Subsequent X-ray structure determinations^5−8^ have corrected the space group (to *C*2/*c* from *Cc*(4)) and show an (almost) *D*_3h_ Fe(CO)_5_ occupying a site of symmetry *C*_2_.

The unusual symmetry has also meant that the vibrational spectroscopy of Fe(CO)_5_ has been extensively investigated since the 1950s.^9−17^ The most comprehensive study was carried out by Jones et al.^16^ who measured the infrared spectra of the ^12^C^16^O, ^13^C^16^O, and ^12^C^18^O isotopomers. Combined with the best Raman data available at the time, they derived a complete force field. Gas phase studies have the advantage that the selection rules are generally rigorously obeyed, so making assignments easier. The disadvantage is that modes that are forbidden in both the infrared and Raman spectra are unobservable. *D*_3h_ Fe(CO)_5_ has one such mode, and this had to be deduced from overtone and combination bands.

Most studies have been of the gas or liquid phase, with comparatively few of the solid state.^13−15^ In the solid state, the low crystal symmetry results in, formally, all the modes being allowed in both the infrared and Raman spectra. In practice, such modes are generally weak and difficult to distinguish from overtone and combination bands.

To overcome the uncertainty in the assignments, there have been many computational studies of Fe(CO)_5_.^18−21^ To date, these have all used the isolated molecule, that is, a gas phase approximation. We are unaware of any calculations of the solid-state vibrational spectra.

Inelastic neutron scattering (INS) spectroscopy^22^ offers an alternative approach. INS is a complementary form of vibrational spectroscopy, whose major advantage for the study of metal carbonyls is that there are no selection rules and all the modes are, in principle, observable. In practice, the resolution in the C≡O stretch region is insufficient to resolve the modes; however, this is the region that has been the most comprehensively studied by infrared and Raman spectroscopies. In the metal carbonyl stretch and deformation region below 800 cm^–1^, the modes are easily resolved. Crucially, this is the region where the infrared and Raman forbidden mode occurs. This method was used to observe all the modes in this region (including the forbidden ones) for the metal hexacarbonyls, M(CO)_6_, M = Cr, Mo, and W.^23^ The assignments were supported by periodic density functional theory (periodic-DFT) calculations of the solid-state structure.

In this work, we have determined the solid-state structure by neutron powder diffraction between 10 and 240 K; the melting point is at 252 K. This work revealed a hitherto unknown phase transition at ∼105 K from the *C*2/*c* phase to a *P*1̅ phase. We note that all the spectroscopic data is from the 1970s or earlier. To update and complement this, we have measured the INS spectra in both phases and recorded Raman spectra in the range 7–300 K, encompassing both solid-state phases and the liquid state. Infrared spectra of the *C*2/*c* phase and the liquid were measured from 50 cm^–1^.

## Results and Discussion

### Neutron Powder Diffraction

**Figure 1:**
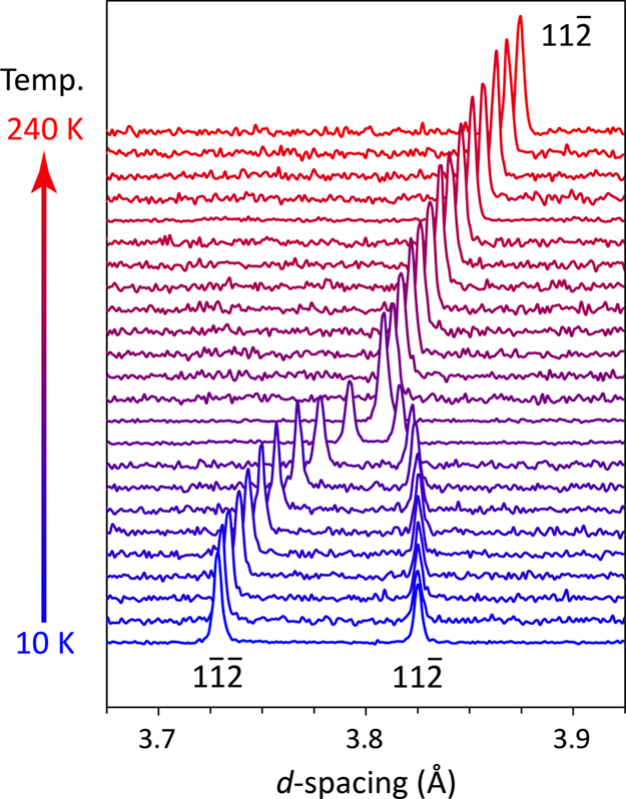
Stack plot of neutron powder diffraction data collected on warming from 10 to 240 K, illustrating the splitting of one of the Bragg peaks below 110 K due to the *C*2/*c* ↔ *P*1̅ phase transition.

Structural models of Fe(CO)_5_ obtained by Rietveld refinement of neutron powder diffraction data measured at 200 and 110 K are in good agreement with the most recent X-ray single-crystal diffraction results^7,8^ (Table 1). We find that the axial Fe–C and C–O lengths are indeed longer and shorter, respectively, than the equivalent equatorial values with little evidence of any significant changes on cooling from 200 to 110 K. However, a splitting of the Bragg peaks in the powder diffraction patterns was observed between 110 and 100 K (Figure 1), indicative of a structural change with a reduction of the crystal’s symmetry from monoclinic to triclinic. There have been no prior reports of a low-temperature phase transformation in Fe(CO)_5_, and the heat capacity data that extend down to 22.59 K exhibit no significant anomalies.^24^ Similarly, the most recent single-crystal study^8^ was carried out at 100 K, just below the temperature at which we observe the transition. These authors reported residual features in their Fourier difference maps that required the implementation of an anharmonic model of the atomic displacements, but otherwise nothing untoward was noted.

**Table 1:** Comparison of Intramolecular Bond Lengths in Fe(CO)_5_ Phase I between Our Work and Values Reported in the Literature

	200 K^a^	200 K^6^	198 K^7^	110 K^a^	100 K^8^
C1–O1	1.156(2)	1.129(8)	1.136(2)	1.147(2)	1.1451(5)
C2–O2	1.111(1)	1.126(9)	1.117(2)	1.122(2)	1.1387(5)
C3–O3	1.131(2)	1.17(2)	1.128(4)	1.130(2)	1.1444(9)
Fe1–C1	1.809(2)	1.805(7)	1.804(3)	1.807(2)	1.8131(3)
Fe1–C2	1.824(1)	1.805(7)	1.811(2)	1.823(1)	1.8187(3)
Fe1–C3	1.814(2)	1.76(1)	1.801(3)	1.816(2)	1.8098(5)

a This work.

Since the observed transition was apparently displacive in nature, with only a lowering of the molecular site symmetry from *C*_2_ down to *C*_1_, it proved straightforward to derive a structural model of the low-temperature phase and carry out refinements against the neutron powder diffraction data measured at 100 and 10 K. The results of these refinements are reported in the Supporting Information using the *P*1̅ cell, but for the purposes of continuity in describing the temperature dependence of the unit-cell parameters, we otherwise adopt the nonprimitive *c*-face-centered triclinic space group, *C*1̅, to characterize the low-temperature behavior. Neutron powder diffraction patterns and fitted profile refinements at 10 K and 200 K are depicted in Figures S1 and S2, respectively.

**Figure 2:**
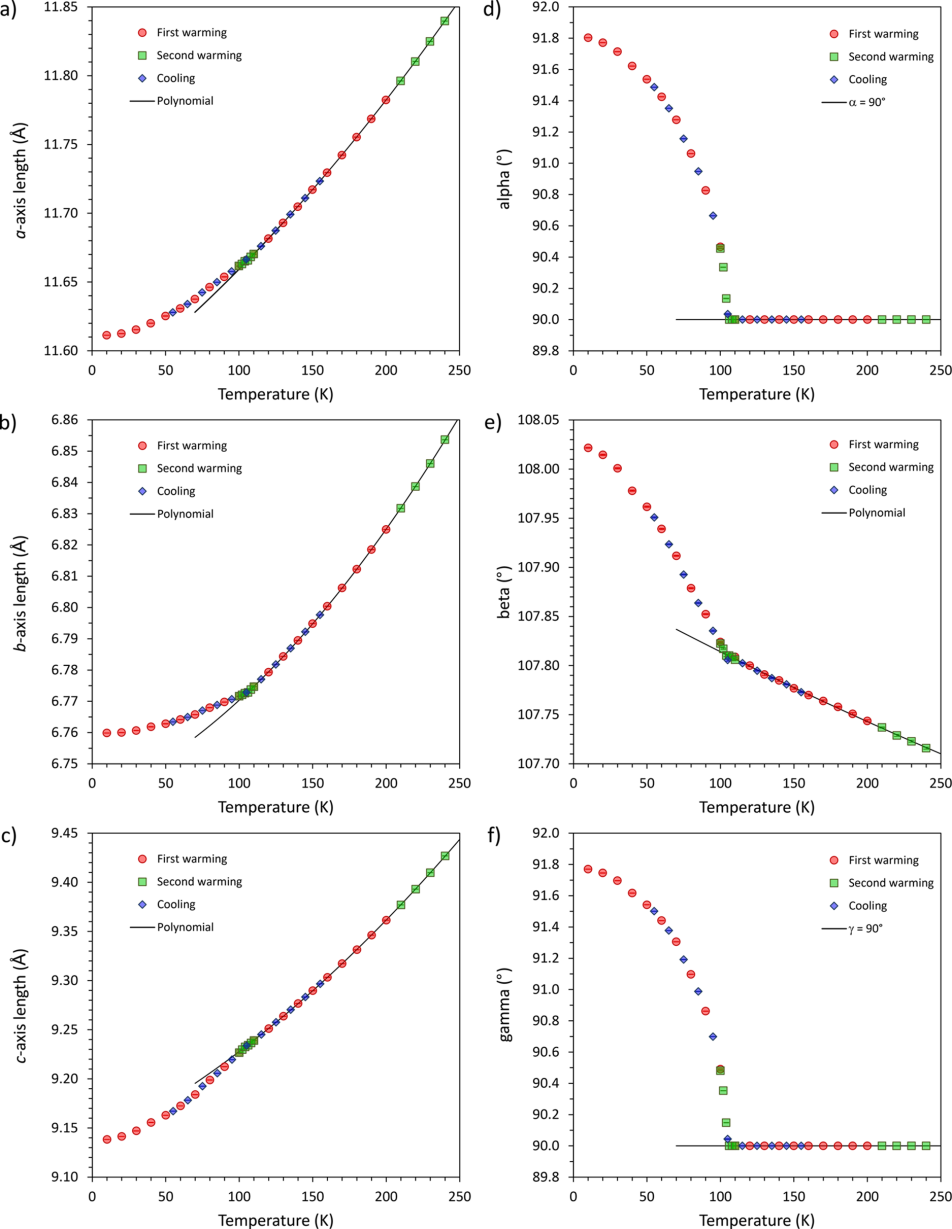
Unit-cell parameters of Fe(CO)_5_ as a function temperature: (a) *a*-axis length; (b) *b*-axis length; (c) *c*-axis length; (d) interaxial angle α; (e) interaxial angle β; and (f) interaxial angle γ. The solid lines are polynomials fitted to the data above the phase transition and extrapolated 30 K below the transition.

**Figure 3:**
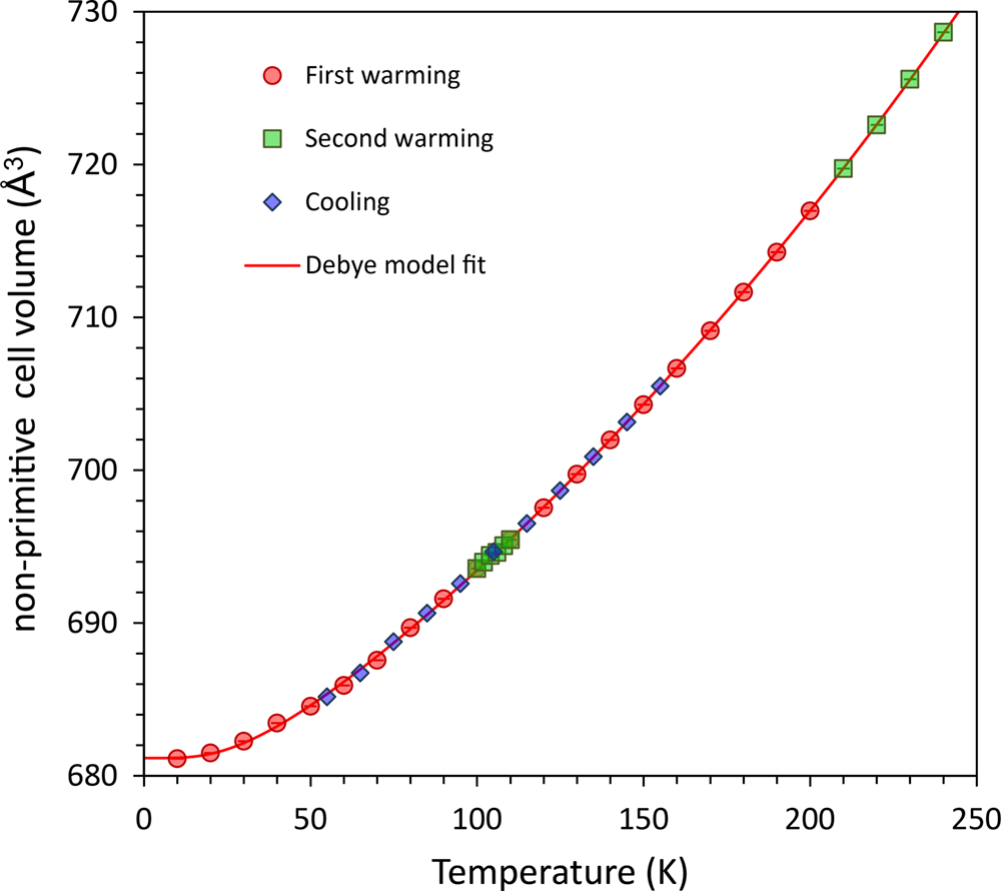
Unit-cell volume of Fe(CO)_5_ as a function temperature. The solid red line represents a Debye-type model of the thermal expansion fitted to the data (see Supplementary Methods).

The unit-cell parameters of the *C*2/*c* and *C*1̅ phases are tabulated in Table S1 and plotted in Figure 2. Inflections in the temperature dependence of these parameters are evident at ∼105 K but are not propagated to the unit-cell volume (Figure 3), where no discontinuity or inflection can be observed. The transition is found to be reversible and reproducible, being observed at the same temperature on cooling and in two separate sequences of data collected on warming with no significant hysteresis.

The distortion of the unit cell due to the *C*2/*c* → *C*1̅ transition, independent of the effect due to changes in temperature, is described as a spontaneous strain, ε_S_.^25^ This strain is a symmetrical second-rank tensor with six independent elements (e_*ij*_), derived from the unit-cell parameters of the high- and low-temperature phases at any given datum.^26^ Clearly, due to the transition, the unit-cell parameters of the high-temperature phase cannot be measured in the region of stability of the low-temperature phase and must be obtained by extrapolation. Fortunately, there are data over a sufficiently wide range of temperatures above the transition for a simple quadratic polynomial expression to be fitted, which may then be extrapolated a few tens of degrees below the transition without introducing significant artifacts. These polynomial fits, and their extrapolations, are shown in Figure 2; the fit parameters are listed in Table S2. From the resulting spontaneous strain tensors, we find that the nonsymmetry-breaking strains (e_nsb_), e_11_, e_22_, e_33_, and e_13_, exhibit a small but linear dependence on temperature, whereas the symmetry-breaking shear strains (e_sb_), e_12_ and e_23_, display a much larger *T*^1/2^ dependence on temperature below the phase transition (Figure S3).

**Figure 4:**
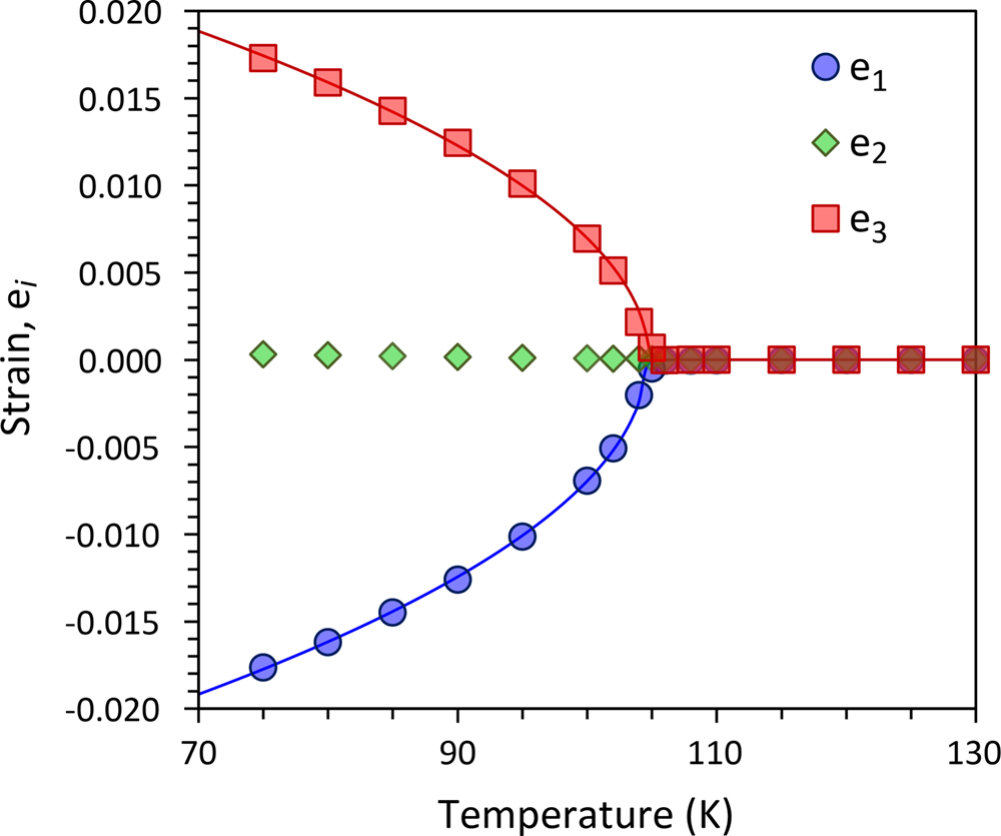
Principal elements of the spontaneous strain tensor fitted with a model assuming thermodynamically second-order behavior (solid lines).

Standard matrix decomposition methods are applied to obtain the eigenvalues and eigenvectors of the spontaneous strain tensor.^27^ These constitute the principal tensile strains, e_1_, e_2_ and e_3_, along three orthogonal axes of the strain ellipsoid and the orientation of the ellipsoid with respect to the original crystallographic reference frame. Figure 4 shows that e_1_ and e_3_ vary with *T*^1/2^ below the transition and are almost perfectly symmetrical, with e_2_ ≈ 0 at all *T*.

The temperature dependence of these terms is typically described using Landau theory, in which the strain is related to one or more transition-driving order parameters (*Q*) that are representative of the thermodynamic character of the transition.^28^ For a thermodynamically second-order phase transition, ε ∝ *Q* ∝ (*T*_C_ – *T*)^1/2^, where *T*_C_ is the critical temperature of the transition. A least-squares fit of the equation e_*i*_ = *x_i_*(*T*_C_ – *T*)^1/2^ to e_1_ and e_3_ in the range 70–100 K yields *T*_C_ = 104.5(1) K from e_1_ and 104.8(2) K from e_3_. Both strains have a near identical degree of coupling to the order parameter: *x*_1_ = 1.07(1) × 10^–5^ and *x*_3_ = 1.02(1) × 10^–5^.

**Figure 5:**
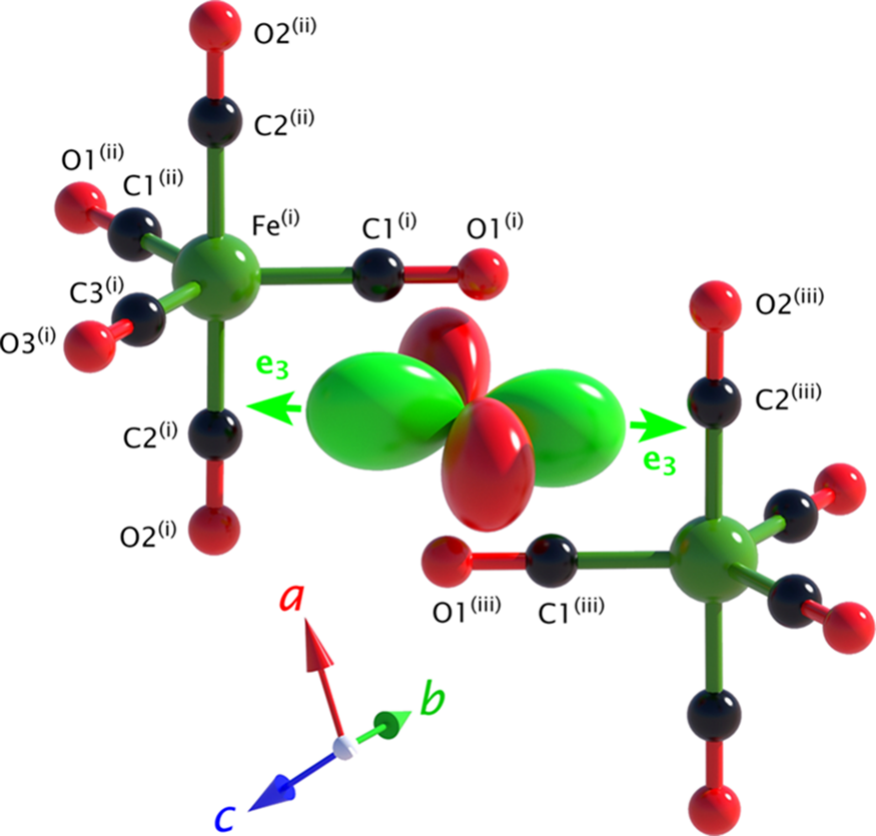
Spatial relationship between the spontaneous strain tensor’s representation surface (lobate figure, centrally positioned) and the local molecular environment. Symmetry codes: (i) *x*, *y*, *z*; (ii) −*x*, *y*, 1/2 – *z*; (iii) 1/2 + *x*, 1/2 – *y*, 1/2 + *z*. Tensor drawn using WinTensor.^30^

Figure 5 shows a tensor representation surface^29^ computed from the e_*ij*_ at 75 K and depicted in relation to the structure of Fe(CO)_5_: lobes colored in green indicate positive tensile strain (expansion) and lobes colored in red connote negative strain (contraction). This arrangement leads to planes of pure shear between the lobes. Alternative views of the representation surface, including comparisons with the thermal expansion tensors, are provided in Figures S4 and S9.

An alternative evaluation of the symmetry-breaking strains may be obtained without recourse to extrapolation. As described by Salje,^31^ when the change in β is small, then e_12_ is simply proportional to cos(γ) and e_23_ ∝ cos(α*). In this instance, we would therefore expect cos^2^(α*) to exhibit a linear temperature dependence, which Figure S5 shows to be the case, with an *a*-axis intercept = 104.6(3) K. Similarly, for the situation where e_12_ and e_23_ are driven by a single order parameter, then we should expect a linear relationship between cos(γ) and cos(α*): Figure S5b confirms this.

In order to interpret the origin of the phase transition, we must characterize the intermolecular distances and interactions. We first employ Hirschfeld surfaces^32^ and their related two-dimensional fingerprint plots^33^ to examine the spatial relationships: these have been computed for Fe(CO)_5_ from the crystal structures determined at 10, 100, 110, and 200 K using CrystalExplorer 17.5.^34^ The distance from a point on the Hirschfeld surface to the nearest nuclei inside the surface, *d*_i_, and the distance to the nearest external nucleus, *d*_e_, is plotted in Figure S6, with colors indicating the proportion of the Hirschfeld surface area at a given distance. We observe that the interactions are dominated by O···O contacts (>50% of the surface area), followed by C···O and O···C contacts. The area devoted to the latter increases only slightly on cooling, at the expense of the area due to O···O interactions. Qualitatively, the distribution of distances becomes less diffuse on cooling; it is particularly apparent that the distributions of all intermolecular interactions are considerably sharper at 10 K than at 200 K. Nevertheless, these changes appear to vary linearly with temperature and do not exhibit a clear signature of the phase transition.

**Figure 6:**
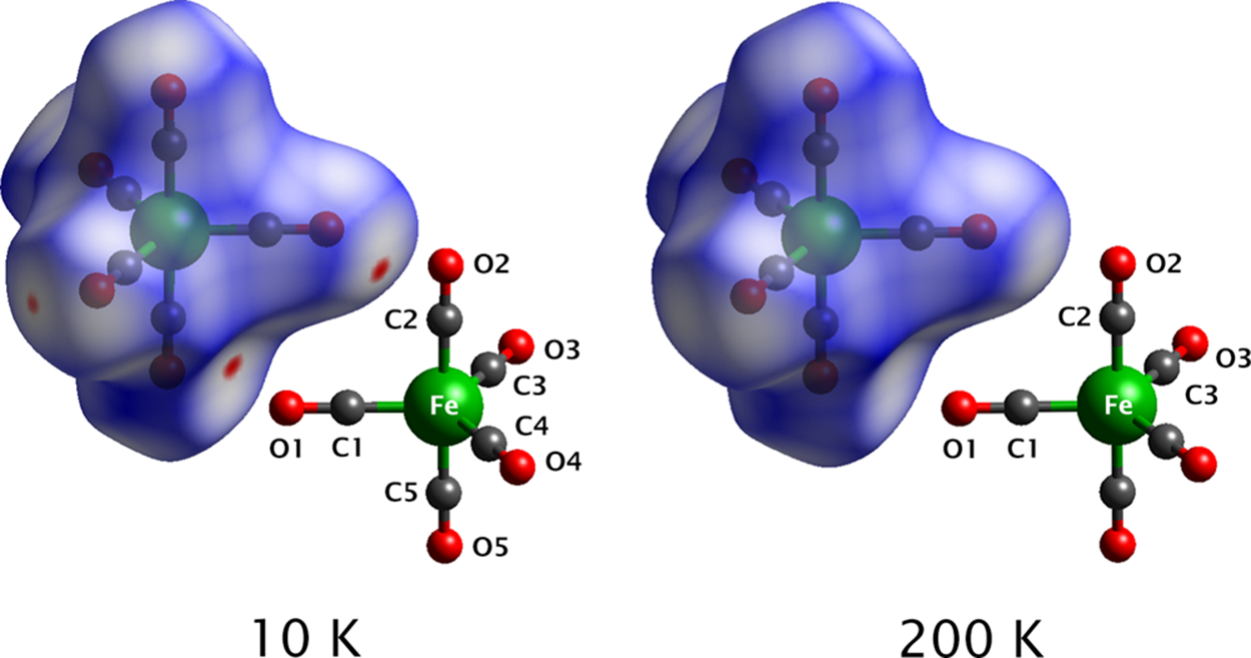
Hirschfeld surfaces at 10 and 200 K, shaded by *d*_norm_ with positive values in blue and negative values in red. The molecular pairs illustrated correspond with the “strong” interactions between molecules “0” and “2” shown in Figure S8a, for which the high relative contribution of exchange-repulsion energy is tabulated in Tables S3 and S5. Note that only the symmetry inequivalent atoms are labeled.

It is useful also to examine the quantity *d*_norm_, in which both d_i_ and d_e_ are normalized by the van der Waals radii for a given pair of atoms and then summed. Hence, for any point on the Hirschfeld surface, a positive value of *d*_norm_ indicates intermolecular contacts that are longer than the sum of the van der Waals radii and negative values indicate contacts shorter than the van der Waals (vdW) sum. The Hirschfeld surfaces at 10 and 200 K are shown in Figure 6, shaded by *d*_norm_ with positive values in blue and negative values in red. Several contacts become shorter than the van der Waals sum on cooling, with distinct red patches appearing on the Hirschfeld surface. For completeness, Hirschfeld surfaces computed at all four temperatures are provided in Figure S7.

**Figure 7:**
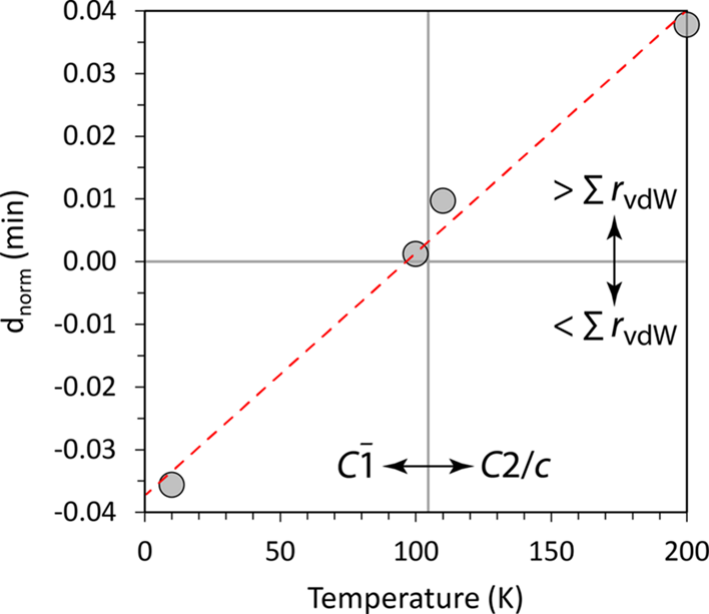
Minimum values of *d*_norm_ on the Hirschfeld surface as a function of temperature.

A plot of the minimum values of *d*_norm_ on the Hirschfeld surface as a function of temperature (Figure 7) reveals that the parameter turns negative very close to the ferroelastic *T*_C_ at ∼105 K. Effectively, the transition occurs when certain interatomic contacts become shorter than their van der Waals sum. Comparing Figures 4 and 5, one can observe that these shortened interactions are operating between the carbonyl group labeled C1–O1 (*x*, *y*, *z*) and C2–O2 (*x* + 1/2, −*y* + 1/2, *z* + 1/2) and their reciprocal pair, C2–O2 (*x*, *y*, *z*) and C1–O1 (*x* + 1/2, −*y* + 1/2, *z* + 1/2). The vectors between these groups correspond with the direction of greatest positive spontaneous strain, e_3_.

We next consider the pairwise interaction energies, which are also obtained using CrystalExplorer 17.5 from wavefunctions computed at the B3LYP-D2/6-31G(d,p) level of theory.^35^ Each iron pentacarbonyl molecule participates in a range of different interactions, illustrated in Figure S8, three of which have a large stabilizing effect (i.e., larger negative total energy, *E*_tot_) with the remaining three being substantially weaker (Table S3). Of the three stronger interactions, it is that with the second nearest neighbor that is of interest from our analysis of the intermolecular distances (cf., Figure 6). Tables S4–S6 show how the individual contributions to the energies of the three strongest interactions vary with temperature, including the ratio of the exchange-repulsion energy (*E*_rep_) to the dispersion energy (*E*_dis_) It is clear, even at 200 K, that the repulsion energy is relatively strong for the second nearest neighbor interaction, approaching equivalence with the dispersion energy at 10 K, which is in striking contrast with the behavior of the other two types of near-neighbor contacts. Furthermore, the repulsion energy appears to drop slightly at the transition, potentially indicating a relief of the strain at the onset of the transition due to the growing repulsion, although it would be beneficial in hindsight to obtain more narrowly spaced structure refinements to confirm this effect. Additionally, the total energy of this interaction becomes less negative as the temperature decreases (*E*_tot_ increases on cooling for the other interactions), showing that this particular interaction becomes less stabilizing in nature.

We interpret these results as follows: the distance between Fe(CO)_5_ molecules shrinks by virtue of thermal contraction, with a degree of anisotropy (Figure S9). As a result, certain intermolecular contacts become shorter than their vdW radii sum, resulting in rising electrostatic repulsion; the relative contribution of this repulsive force is apparently sufficient above some critical threshold to generate a strain that breaks the symmetry of the crystal. Hence, we interpret the displacive ferroelastic transition as being likely due to van der Waals strain. Since the repulsion increases continuously, the enthalpy of the crystal also varies continuously, which conforms with the second-order nature of the transition. The lack of any apparent signature of the phase transition in the heat capacity data^24^ shows that any change in the temperature dependence of the enthalpy must be rather small.

### Vibrational Spectroscopy

In the gas phase, the *D*_3h_ symmetry of Fe(CO)_5_ results in four C**≡**O stretch modes [2***A***_**1**_^′^ (ν_1_, ν_2_), ***A***_**2**_^″^ (ν_6_), ***E***^′^ (ν_10_)], four Fe–CO stretch modes [2***A***_**1**_^′^ (ν_3_, ν_4_), ***A***_**2**_^″^ (ν_8_), ***E***^′^ (ν_13_)], six Fe–C**≡**O bending modes [***A***_**2**_^′^ (ν_5_), ***A***_**2**_^″^ (ν_7_), 2***E***^′^ (ν_11_, ν_12_), 2***E***^″^ (ν_16_, ν_17_)], and four OC–Fe–CO bending modes [***A***_**2**_^″^, (ν_9_), 2***E***^′^, (ν_14_, ν_15_), ***E***^″^ (ν_18_)] (counting doubly degenerate modes, i.e., ***E***^′^ and ***E***^″^, as a single mode. The mode numbering is that of Bigorgne^14^ and Jones et al.^16^). ***A***_**2**_^″^ and ***E***^′^ are infrared-allowed; ***A***_**1**_^′^, ***E***^′^, and ***E***^″^ are Raman-allowed and ***A***_**2**_^′^ is inactive in both the infrared and Raman spectra. In the solid state, the usual approach is the correlation method;^36^ however, the low site symmetry of *C*_2_ and *C*_1_ in the *C*2/*c* and *P*1̅ phases, respectively, means that all degeneracies are lifted and all modes are allowed. The presence of two molecules in the primitive cell of each phase results in every mode having an in-phase and an out-of-phase combination, one of which is infrared-allowed and one Raman-allowed. Thus, the selection rules are the same in both phases. Note that all modes are allowed in the INS in both phases. Our structural study shows that the molecular structure is essentially the same in both phases; see Table S8. Consequently, the spectra in both phases look very similar, and this explains why previous spectroscopic studies did not detect the phase change.

**Figure 8:**
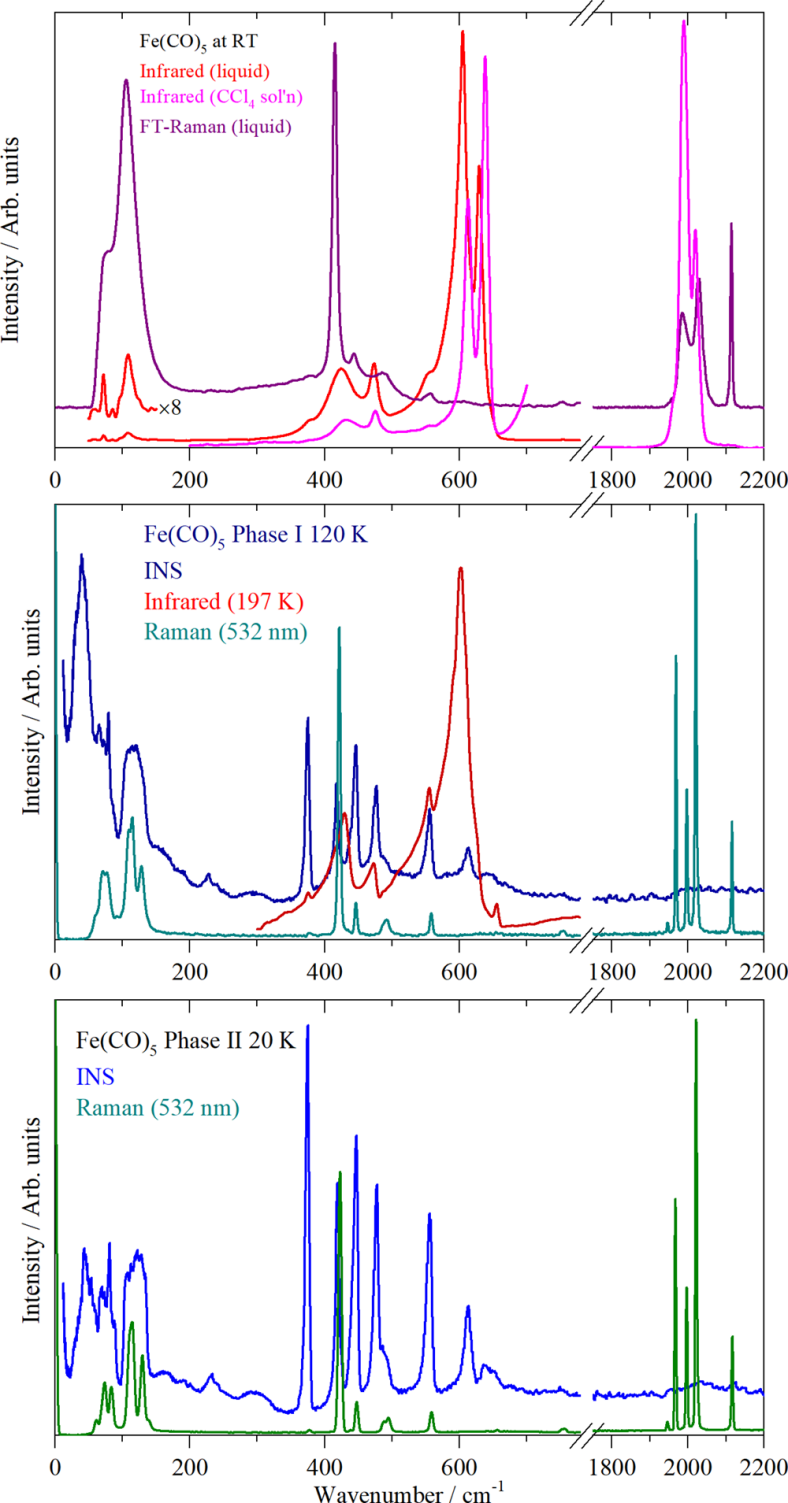
Vibrational spectra of Fe(CO)_5_. Top: liquid at room temperature, middle: solid, phase I (INS and 532 nm Raman at 120 K, infrared at 197 K), bottom: solid, phase II at 20 K.

Figure 8 shows the vibrational spectra of Fe(CO)_5_ in the liquid and two solid phases, and the observed bands are given in Table 2. We defer a detailed assignment of the spectra to the next section, where we support these with periodic-DFT calculations. However, several points are worth noting.

**Table 2:** Observed Bands of Fe(CO)_5_ (cm^–1^) and Their Assignments^a^

liquid at RT			phase I			phase II		assign
infrared	infrared CCl_4_ sol’n	FT-Raman	infrared (197 K)	Raman (120 K)	INS (120 K)	Raman (20 K)	INS (10 K)	
		2114		2116 m		2117 m		ν_1_
		2028		2021 vs		2021 vs		ν_2_
	2020							ν_6_
	1989	1985		1997 m		1997 m		ν_10_
				1969 s		1967 s		ν_10_
				1947 vw		1946 vw		
						756 vw	752 vw	2 × ν_5_
						753 vw		2 × ν_5_
			656 vw		647 w,br	657 vw	655 w	ν_7_
630	639						637 w	ν_7_
605	613		602 vs		612 m		613 m	ν_11_
557	557	557	556 w	559 w	556 s	559 w	556 s	ν_12_
		488		493 w	490 sh	496 w	492 sh	
				487 sh		489 w		
474	476		473 m		476 s		478 s	ν_13_
		443		447 w	447 s	447 w	447 s	ν_3_ and ν_16_
425	431	416	430 m	422 vs	418 s	423 vs	419 s	ν_4_
378			376 vw		375 s	378 vw	375 vs	ν_5_ and ν_17_
				128 m	132 sh	130 m	129 m	ν_18_
							122 m	ν_18_
				116 s		115 m	114 m	ν_14_
108		106		110 sh	110 s, br	110 sh	107 m	ν_14_
							89w	
		78		78 m	78 m	81 m	84 m	ν_15_
72				71 m		74 m	72 m,br	ν_9_
				61 sh	66 w	62 w	59 sh	lattice mode
							54 w	lattice mode
					41 vs,br		45 m,br	lattice mode

a s = strong, m = medium, w = weak, v = very, br = broad, sh = shoulder.

In the 300–700 cm^–1^ region, there are 10 modes. In the INS spectrum of phase II, there are only seven modes apparent. As all modes are allowed and must be present, it immediately follows that there are three accidental degeneracies.

In the region below 200 cm^–1^, in addition to the four OC–Fe–CO bending modes, there are six librational, three optic translational, and three acoustic translational modes. This results in the dense manifold of modes seen in the INS spectra. As the translations require the entire molecule to move, these are likely to form the lowest energy feature, with the librations (which only involve the carbonyl ligands moving) largely comprising the middle feature and the internal modes forming the intense, broad feature centered at 120 cm^–1^. The width of the features would suggest that there is significant vibrational dispersion present (variation of transition energy with wavevector; INS is sensitive to all wavevectors, in contrast to infrared and Raman spectroscopy that are seen at zero wavevector). This is noticeably distinct from the 300–700 cm^–1^ region, where the bands are nearly resolution-limited, indicating almost no dispersion is present.

### Computational Studies and Assignment of the Spectra

**Figure 9:**
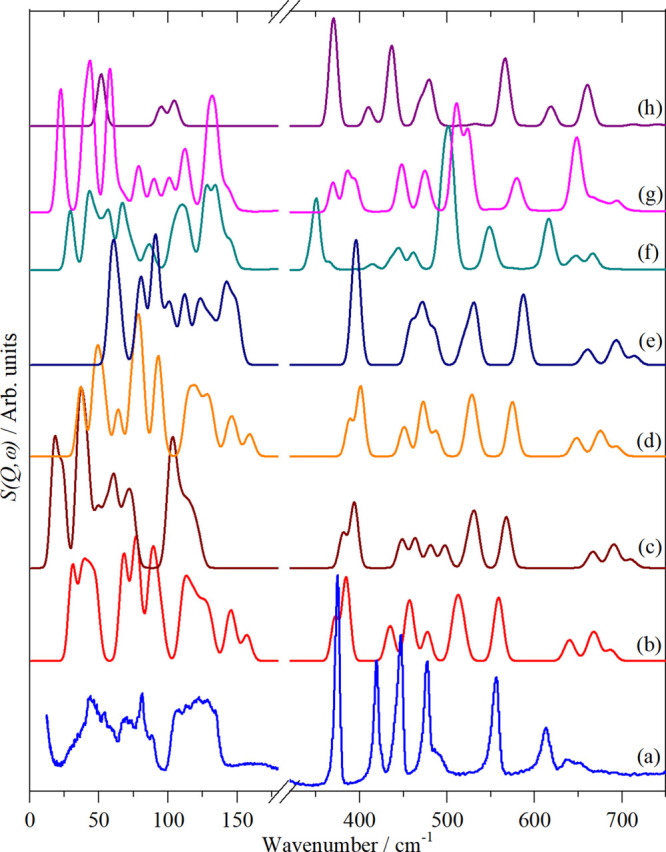
Comparison of observed and calculated (from DFT) spectra of Fe(CO)_5_. (a) Experimental spectrum at 20 K (phase II), (b) CASTEPv17 GGA (PBE, TS, OTFG), (c) CASTEPv17 LDA (CA-PZ, OBS, OTFG), (d) CASTEPv17 GGA (PBE, TS, OPIUM), (e) CASTEPv20 GGA (rSCAN, OTFG), (f) DMol3 (BLYP, DNP), (g) DMol3 (SCAN, DNP), and (h) Gaussian09 (B3LYP, aug-ccVTZ). See the Computational Studies section of the Experimental section for the definition of the acronyms.

Comparison of an experimental INS spectrum with that generated from a DFT calculation is a well-established method for the assignment of vibrational spectra.^22^ It was used to assign the solid-state spectra of the metal hexacarbonyls, M(CO)_6_, M = Cr, Mo, W.^23^ The agreement between observed and calculated spectra was very good and enabled unambiguous assignments to be made. Our expectation was that the same would also be true for Fe(CO)_5_. As Figure 9 shows, this is not the case.

The calculations used a variety of methods and programs. CASTEP (Figure 9b–e) is a periodic method that uses plane-waves, DMol3 (Figure 9f,g) was used in its periodic implementation with atom centered orbitals, and Gaussian09 (Figure 9h) is an isolated molecule calculation that uses atom centered orbitals. The calculated geometry (Table S2) generally showed good agreement with the experimental data. Several different functionals and types of pseudopotential were used, and it is apparent that none are completely satisfactory.

However, there are common features between the calculations, and together with our new data, this is sufficient for a definitive assignment of the solid-state spectra of Fe(CO)_5_.

Table 3 summarizes the previous assignments. In the C≡O stretch region, there is general agreement on the assignments. In the gas and liquid phases, three Raman bands and two infrared bands are expected and observed. The selection rules make the assignments straightforward. The two polarized bands in the Raman that do not have infrared counterparts are the *A*_1_^′^ modes ν_1_ and ν_2_; the infrared band without a Raman counterpart is the *A*_2_^″^ mode ν_6_ and the lowest energy mode that occurs in both the infrared and Raman spectra is the *E*^′^ mode ν_10_. In the solid state, four strong bands are seen in the Raman spectrum. The two highest energy bands are the *A*_1_^′^ modes; the lowest pair were assigned as the *E*^′^ mode with the degeneracy lifted by the crystal symmetry.^15^ The calculations support this interpretation. The possibility that one of the modes is the Raman-allowed component of the gas phase *A*_2_^″^ infrared-only mode that has become active in the solid state can be discounted because the calculated intensity is very low.

**Table 3:** Fe(CO)_5_ Assignments (cm^–1^)

sym	mode no.	description	Edgell^13^	Bigorgne^14^	Catalotti^15^	Jones^16^	Delley^18^	Jonas^19^	Schaefer^20^	this work
			Expt.	Expt.	Expt.	Expt.	Calc.^a^	Calc.^b^	Calc.^c^	
			gas	liquid	solid	gas				solid
*A*_1_^′^	ν_1_	ν(C≡O)	2117	2116	2115	2121	2072	2090	2169	2117
*A*_1_^′^	ν_2_	ν(C≡O)	1984	2030	2033	2042	1991	2012	2093	2021
*A*_1_^′^	ν_3_	ν(Fe–CO)	414	418	410	443	441	453	439	447
*A*_1_^′^	ν_4_	ν(Fe–CO)	377	381	440	413	419	428	413	419
*A*_2_^′^	ν_5_	δ(Fe–C≡O)		379	593	383	349	361	364	375
*A*_2_^″^	ν_6_	ν(C≡O)	2014	2022	2003	2034	1989	2011	2094	2020
*A*_2_^″^	ν_7_	δ(Fe–C≡O)	620	615	623	619	610	621	617	655/637
*A*_2_^″^	ν_8_	ν(Fe–CO)	474	430	433	429	480	485	473	427
*A*_2_^″^	ν_9_	δ(OC–Fe–CO)		72		[100]^e^	105	104	107	72
*E*^′^	ν_10_	ν(C≡O)	2034	2000	1980	2013	1974	1990	2067	1997
*E*^′^	ν_11_	δ(Fe–C≡O)	646	642	643	645	646	657	660	613
*E*^′^	ν_12_	δ(Fe–C≡O)	544	553	558	542	479	489	483	556
*E*^′^	ν_13_	ν(Fe–CO)	431	475	480	474	424	436	439	478
*E*^′^	ν_14_	δ(OC–Fe–CO)	104	114		105	103	100	104	106
*E*^′^	ν_15_	δ(OC–Fe–CO)	68	64		[74]^e^	54	50	54	84
*E*^″^	ν_16_	δ(Fe–C≡O)	752	488	487	488	539	552	563	447
*E*^″^	ν_17_	δ(Fe–C≡O)	492	448	614	[375]^e^	363	374	366	375
*E*^″^	ν_18_	δ(OC–Fe–CO)	95	130^d^		[97]^e^	94	93	95	129/122

a B88-LYP with a double numerical basis set.

b BP86 with an ECP2 basis set.

c B3LYP with a double-ζ plus polarization (DZP) basis set.

d Solid state.

e Estimated from combination bands.

The assignment of the 10 modes in the 300–700 cm^–1^ region has been controversial. Table 3 lists the assignments, and it can be seen that there are significant disagreements. The assignments fall into two camps: those based on experimental work^13−16^ and those based on computational studies.^18−20^ There is general agreement within each group, except for the assignments of Edgell,^13^ which were based on incomplete data. The computational studies reversed several assignments, for example, ν_8_ and ν_13_ and ν_12_ and ν_16_, but these were done largely to obtain better agreement with the experimental data. As we have demonstrated in Figure 9, computational studies provide a poor description of the experimental spectra.

Some of the difficulties arise because the selection rules are less helpful than expected. These only predict whether a mode is infrared- or Raman-allowed but have nothing to say about its intensity. A mode may be allowed but have negligible intensity and this is the case with several modes here. The presence of accidental degeneracies further complicates the problem.

Two modes for which symmetry does deliver clear evidence are ν_3_ (*A*_1_^′^) and ν_12_ (*E*′). Polarization measurements^12,14,16^ show the intense Raman line at 416 cm^–1^ to be strongly polarized, so it must be an *A*_1_^′^ mode, that is, ν_3_. The band at 556 cm^–1^ is clearly present in both the infrared and Raman spectra of the liquid, so it must be an *E*′ mode.

The calculations do provide some useful information. All the calculations predict the “forbidden” mode ν_5_ to be close in energy to the *E*^″^ mode ν_17_ and both to be ∼380 cm^–1^. To a first approximation, the INS intensity of an *E* mode will be twice that of an *A* mode for the same type of motion. This is seen in Figure 9b–d, and it is apparent that to account for the intensity of the 380 cm^–1^ INS mode, the two modes must be accidentally degenerate.

Some time ago we showed^37^ that, provided that the geometry was reasonably accurate, the mode eigenvectors that describe the motion (i.e., the amplitude of vibration of each atom in the mode) are relatively insensitive to the eigenvalue (transition energy). This means that the calculated transition energies can be shifted to match the experimental values as a means to test an assignment scheme. Table S9 shows that the bond distances are within 0.03 Å, and the angles are within 0.5°, thus meeting the structural accuracy criterion.

**Figure 10:**
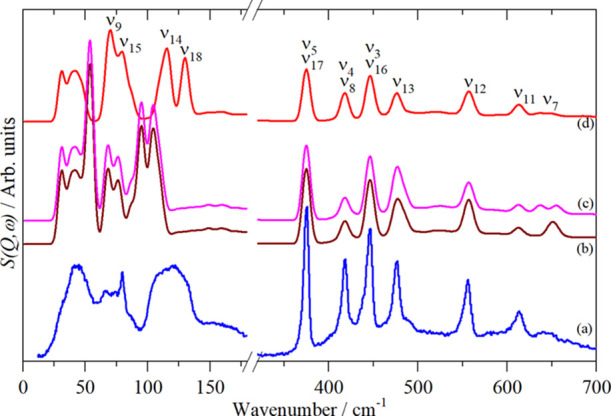
Observed and calculated INS spectra of Fe(CO)_5_: (a) phase II at 20 K, (b) simulation of the literature assignments,^18−20^ (c) the same as (b) but with the degeneracy removed from ν_11_ and (d) with final assignments (see Table 3).

Figure 10b shows the calculated spectrum based on the experimental assignments (columns 5 and 7) and Figure 10c based on the computational assignments (columns 8–10) given in Table 3. To generate the calculated spectra, we have used one of the CASTEP calculations (that shown in Figure 9b) and shifted the internal modes to the predicted positions, leaving the lattice modes unchanged. We have also made the assignment ν_5_ = ν_17_ = 375 cm^–1^.

The strong infrared Fe–C≡O bending modes, ν_7_ (*A*_2_^″^) and ν_11_ (*E*^′^), have been assigned to the bands at 613 and 639 cm^–1^. This assignment is based on the reported^14^ presence of a very weak band in the Raman spectrum of the liquid at 653 cm^–1^. A subsequent study^16^ did not detect this band, and we do not observe it in the liquid. There is a weak band at 657 cm^–1^ in the solid-state Raman spectra and bands at 613, 637, and 657 cm^–1^ are clearly seen in the INS spectra. Simulating the INS spectrum with the literature assignments generates the spectrum shown in Figure 10b. Comparison with the experimental spectrum (Figure 10a) shows that the relative intensities of the modes in the 600–700 cm^–1^ region are inverted. If the degeneracy of ν_11_ is lifted and the components assigned to the 637 and 657 cm^–1^ bands, as can be seen from this region in Figure 10c, there is still an intensity mismatch. If the assignments are reversed, that is, ν_11_ (*E*^′^) = 613 cm^–1^ and the two factor group components of ν_7_ (*A*_2_^″^) are assigned at 637 and 657 cm^–1^, as seen in Figure 10d, there is good agreement with the experimental data, as shown in Figure 10a. ν_7_ is the only mode that shows a significant factor group splitting in this region; the reasons for this are unexplained.

At this stage, 6 of the 10 modes have been assigned; the remaining 4 are 3 Fe–CO stretch modes [*A*_1_^′^ (ν_3_), *A*_2_^″^ (ν_8_), *E*^′^ (ν_13_)] and 1 Fe–C≡O bending mode (*E*^″^ (ν_16_). In the spectra, features at 427 and 475 cm^–1^ (infrared) and 447 and 489 cm^–1^ (Raman) are present. In the INS, there is a “trident” of strong modes at 420, 447, and 477 cm^–1^ and a weak feature at 490 cm^–1^. Previous work has assigned the infrared mode at 475 cm^–1^ to ν_13_, with the 490 cm^–1^ Raman band considered to be its Raman counterpart. In the solid, the 490 cm^–1^ band is resolved into two components, as shown in the bottom part of Figure 8, at 488 and 495 cm^–1^. Bigorgne^14^ assigned the lower energy one to ν_13_ and the higher energy one to ν_16_ (*E*^″^). “For lack of better evidence”, Jones et al.^16^ also adopted this assignment. The INS data show that this cannot be correct. There is an intense band at 477 cm^–1^ coincident with the infrared mode, so this must be ν_13_ (*E*^′^), but there is insufficient intensity for the INS feature at 490 cm^–1^ to be an *E* mode. We will return to the assignment of this mode later.

By default, the infrared band at 427 cm^–1^ must be ν_8_ (*A*_2_^″^). Based on a matrix isolation study,^17^ the assignment of ν_8_ and ν_13_ was subsequently reversed and this has been adopted in the computational studies.^18−20^ The justification was that under a specific set of conditions, the 477 cm^–1^ band was present as a doublet, while the 427 cm^–1^ band was always a singlet, suggesting that they were *E* and *A* modes, respectively. The INS shows that this is not tenable; there is insufficient intensity in an *A*-type mode to account for the INS band at 477 cm^–1^. We note that the matrix infrared spectra show the presence of multiple sites on deposition; that more than one can persist is not unreasonable.

The only remaining Raman mode is the feature at 447 cm^–1^ and Jones^16^ has provided convincing arguments that this must be ν_3_ (*A*_1_^′^). There is insufficient intensity in an *A* mode to account for the strength of the 447 cm^–1^ INS band, so this must be coincident with the *E*^″^ mode, ν_16_. The latter has also been assigned to the band at ∼550 cm^–1^ by the computational studies,^18−20^ with ν_12_ at 447 cm^–1^. This is not credible because there is a band in the gas phase infrared spectrum^16^ at 542 cm^–1^ and the intensity is incompatible with it being an overtone or combination, so it must be a fundamental. Recall that *E*^″^ modes are infrared-inactive.

The Raman and infrared spectra show ν_4_ and ν_8_ at 419 and 427 cm^–1^, respectively. To account for the intensity of the INS band at 419 cm^–1^, ν_8_ must be accidentally degenerate with ν_4_ in the solid state. With these assignments, good agreement between the observed and calculated INS spectra is obtained, as shown in Figure 10a,d.

The lattice modes (translations and librations) and the four OC–Fe–CO bending modes occur in the region below 150 cm^–1^. In the gas phase infrared spectrum,^16^ the only mode detected is at 105 cm^–1^. In the liquid, we find weak infrared bands at 72 and 108 cm^–1^, and in the Raman spectrum, we find strong bands at 80 and 106 cm^–1^. The infrared–Raman coincidence shows that the ∼106 cm^–1^ band must be the *E*^′^ mode ν_14_. The 72 cm^–1^ band must therefore be ν_9_, (*A*_2_^″^). This band was also found by Bigorgne^14^ but not by Jones,^16^ who assumed ν_9_ must be in the vicinity of ν_14_ and hence assigned it as 100 ± 15 cm^–1^.

The intense liquid phase Raman band at 106 cm^–1^ is unusually broad and the reason for this becomes apparent in the solid state, where a strong mode is revealed at ∼130 cm^–1^. This was also seen by Bigorgne^14^ but has been overlooked by everyone else. We assign this as the *E*^″^ mode ν_18_.

The remaining liquid phase Raman band at 80 cm^–1^ is therefore the second *E*^′^ mode ν_15_, seen in the solid state at 84 cm^–1^. This provides a ready explanation for the 490 cm^–1^ INS band and the 488/495 cm^–1^ Raman doublet as the combination (ν_15_ + ν_4_) with symmetry *A*_1_^′^ ⊗ *E*^′^ = *E*^′^, hence Raman-active.

In the low energy region, the congested nature of the INS spectrum makes it less useful for assignments. However, the intensity and width of the feature at ∼120 cm^–1^ is consistent with our assignment of two *E* modes being present.

Figure 10d shows the INS spectrum generated using the assignments in the last column of Table 3. It can be seen that the positions and relative intensities of the modes in the 350–700 cm^–1^ region are well reproduced. The profile in the region below 200 cm^–1^ is approximately correct but the intensity relative to the 350–700 cm^–1^ region is too high. In part, this is because the simulated spectrum is based on a Γ-point-only calculation, whereas the INS spectrum is sensitive to all wavevectors across the entire Brillouin zone, so the vibrational dispersion will broaden the modes. The low site symmetry and close proximity in energy also means that mode coupling will be significant, which means that the factor group splitting is much larger than in the higher energy region. In particular, this probably accounts for why the INS feature at ∼120 cm^–1^ is so ill-defined.

## Conclusions

In this work, we have re-examined the structure and vibrational spectroscopy of Fe(CO)_5_, both of which have provided surprises. The diffraction study found a hitherto unknown phase transition, although a recent study^38^ of iron carbonyl using Raman spectroscopy had determined that two high-pressure phases exist in the region up to ∼16 GPa and ∼600 K, above which pressures and temperatures the material breaks down into a mixture of hematite and a polymeric C−O solid. In that work, the I → II phase boundary was represented by a straight line with a zero-pressure intercept at ∼160 K; however, the scatter of their observations is consistent with a steeper d*T*/d*P* at low pressure that could result in an intercept closer to 100 K. It thus seems reasonable to hypothesize that the high-pressure phase II is the same as our low-temperature phase II. This is supported by Raman mode splitting at the high-pressure I → II transition, indicating a lowering of the molecular symmetry. A high-pressure diffraction study is required to further test this hypothesis.

Our diffraction study has shown that in the solid state, the axial Fe–C and C≡O lengths are longer and shorter than the equivalent equatorial values, at all temperatures from just below the melting point at 252 to 10 K, as shown in Table S8. In agreement with earlier GED studies,^2^ the most recent GED study^39^ found the axial Fe–C bond lengths to be shorter than the equatorial ones, while the C≡O bond lengths were equal within errors. There appears to be a real difference between the solid and gas phase structures.

Our work reports the characterization of a ferroelastic transition using high-resolution neutron powder diffraction. Analyses of such phenomena in organic or metal–organic crystals are relatively uncommon and usually confined to high symmetry systems and transitions closer to room temperature. Furthermore, it is usual for strain arising from such transitions to be determined using mechanical gauges rather than diffraction. In this instance, we have evaluated the strain in a triclinic crystal, requiring a description of the spontaneous strain in terms of a second-rank tensor, something that is straightforward to achieve with high-precision powder diffraction data but practically very difficult using strain gauges on a single crystal of such low symmetry. In addition, the diffraction data permit a determination to be made of subtle changes in the crystal structure that, via a Hirschfeld surface analysis, suggest to us that the origin of the transition is van der Waals strain. There is considerable interest in developing molecular strain gauges based on ferroelastic-layered van der Waals solids^40^ and the phenomenological insights garnered through this study may guide the design of materials with real-world applications.

INS spectroscopy has enabled the observation of the internal modes for the first time. While only one mode is forbidden in the gas phase, there are several other modes that were only known from overtones and combinations. The unique attribute of INS spectroscopy that the intensity is determined largely by the amplitude of motion, which means that it has been possible to test the various assignment schemes. All previous assignment schemes are incorrect in, at least, several respects. The assignment in the last column of Table 3 is the only one that is compatible with all the information from infrared, Raman, and INS spectroscopies.

The biggest surprise in this work is the failure of DFT to correctly predict the vibrational spectra. Previous work on the metal hexacarbonyls^23^ yielded calculated spectra in excellent agreement with the experimental data. As shown in Figure 9, this is not the case for Fe(CO)_5_. This also explains why most of the revisions to the assignments made on the basis of the computational studies must be rejected. Why DFT fails here is difficult to understand. The calculated geometry, as shown in Table S9, has bond lengths that differ from experimental values by less than 0.03 Å and the angles by less than 0.5°. For comparison, the accuracy of the calculated geometry of the metal hexacarbonyls^23^ was similar.

## Supporting Information Available

Experimental methods, lattice parameters as a function of temperature, comparisons of observed and calculated neutron diffraction patterns, representation surface of the spontaneous strain tensor, temperature dependence of the reciprocal lattice parameter, Hirschfeld surfaces, symmetry inequivalent interactions between near-neighbor Fe(CO)_5_ and their energies, thermal expansivities, bond lengths and angles of Fe(CO)_5_ as a function of temperature, and comparison of experimental structures (PDF)
