# Supplementary material for: Structure and Spectroscopy of Iron Pentacarbonyl, Fe(CO)5
Source: J Am Chem Soc. 2022 Sep 16;144(38):17376–86. doi: 10.1021/jacs.2c01469 (PMC9523711; doi:10.1021/jacs.2c01469)
Supplement: Supplementary file 1 — ja2c01469_si_001.pdf [file ja2c01469_si_001.pdf]

Electronic Supplementary Information

For:

Structure and spectroscopy of iron pentacarbonyl, $Fe(CO)_5$

A. Dominic Fortes[a] and Stewart F. Parker*[a]

[a]ISIS Neutron and Muon Facility, STFC Rutherford Appleton Laboratory, Harwell Science and Innovation Campus, Chilton, Oxfordshire, OX11 0QX, UK.

E-mail: stewart.parker@stfc.ac.uk

EXPERIMENTAL SECTION

Materials. Iron pentacarbonyl, $Fe(CO)_5$ (99.99%), was purchased from Aldrich and used as received.

Neutron powder diffraction. $Fe(CO)_5$ was frozen by immersion in liquid nitrogen and ground to a fine powder. The powder was transferred into a chilled Al-alloy frame surrounding a cuboid cavity of dimensions 18 x 23 mm (w x h) perpendicular to the incident neutron beam and 15 mm depth parallel to the beam. The front and back faces of the sample were covered with 125 μm-thick vanadium-foil windows, sealed to the Al-frame with indium wire. The sample can was mounted in a closed-cycle refrigerator (CCR) mounted in the sample tank of the High-Resolution Powder Diffractometer (HRPD)[1,2] at the ISIS Neutron and Muon Spallation Source.[3] Cooling of the sample via a bath of helium gas (at ~ 50 mb) was balanced by direct heating of the sample holder using a cartridge heater inserted into the Al-frame. Temperatures were measured and controlled by means of a RhFe thermometer also inserted in the frame on the opposite side of the sample. Previous experience[4,5] has shown that thermal equilibrium between the heated frame and the powder samples during a variable-temperature study requires moderately slow ramping between temperature set-points, ~ 3 K min^{-1}, and a wait of at least 10 m after reaching the set-point prior to commencing data acquisition. After an initial check at 80 K the sample temperature was reduced to 10 K and high-quality data were collected using the instrument's 30–130 ms and 100–200 ms time-of-flight (TOF) windows. In HRPD's highest resolution backscattering detectors (2θ = 154–176°, $\Delta d/d \approx 1.0 \times 10^{-3}$) these two windows cover d-spacing ranges of 0.65 – 2.60 Å and 2.2 – 4.0 Å, respectively. The two datasets were counted for 200 and 160 μA of integrated beam current, respectively. Data were then collected on warming in 10 K increments using only the 100–200 ms TOF window, counting each for 20 μA (~25 m of real time). The more rapidly measured datasets were obtained for the purpose of lattice parameter refinement rather than structure refinement. An additional 'structural' dataset (30–130 and 100–200 ms TOF windows measured for 200 and 160 μA) was acquired on reaching 200 K. Since it was apparent that a phase transformation occurred between 100 and 110 K, further data were collected on cooling. Data were interleaved between the points measured on warming, starting at 155 K and cooling in 10 K increments to 55 K. These data were measured in the 100–200 ms TOF window for 20 μA each. The sample was then warmed to 100 K for the acquisition of a 'structural' dataset just below the transition, shorter measurements on warming in 2 K increments to 110 K and collection of a 'structural' dataset at 110 K, just above the transition. Finally, a few 20 μA measurements were made in 10 K steps from 210 to 240 K to approach the melting point of the sample as closely as time allowed.

These data were time-focussed to a common scattering angle (2θ = 168.3°), normalized to the incident spectrum and corrected for detector efficiency by reference to a V:Nb standard using the Mantid suite of neutron scattering utilities.[6,7] Structural refinements were then carried out using the Rietveld method implemented in GSAS/Expgui[8,9] starting from one of the previously-reported high-

temperature structures of $Fe(CO)_5$.[10] The crystal structures of $Fe(CO)_5$ phase I at 200 and 110 K, and the structures of $Fe(CO)_5$ phase II at 10 and 100 K are provided as supplementary Crystallographic Information Files (CIFs) and have been deposited with the Cambridge Crystallographic Data Centre. Unit-cell parameters obtained from profile refinements done with the *F*(calc) weighted method in GSAS are reported in Table S1.

Vibrational spectroscopy. For the INS measurements ~15 g of $Fe(CO)_5$, was loaded into an In wire-sealed Al can. The sample was quenched in liquid nitrogen immediately before insertion into the indirect geometry, high resolution, broad band spectrometer TOSCA[11,12] at ISIS.[3] TOSCA also has a diffraction capability and this was used to confirm that the sample was in the triclinic phase at 10 K. After measurement at ~10 K, the sample was heated to 120 K to measure the monoclinic phase (also confirmed by neutron diffraction).

Raman spectra of the liquid in a quartz cell and after freezing in liquid nitrogen were recorded using a Bruker FT-Raman spectrometer (64 scans at 1 cm^{-1} resolution with 500 mW laser power at 1064 nm). Variable temperature (7 – 300 K) Raman spectra were recorded with a previously described,[13] Renishaw in-Via system using 532 nm excitation.

Infrared spectra (64 scans at 4 cm^{-1} resolution with eight times zerofilling (to improve the peak shape)) were recorded with a Bruker Vertex 70 FTIR spectrometer. Room temperature spectra of the liquid over the range 50-4000 cm^{-1} were obtained using the Bruker Diamond ATR accessory and in transmission over the range 350-4000 cm^{-1} as a CCl_4 solution in a KBr cell. Spectra (300-4000 cm^{-1}) of the monoclinic phase at 170 K were recorded using a SpecAc Golden Gate variable temperature accessory.

Computational studies. DFT calculations were carried out using three different codes: CASTEP[14] (v17 and v20), DMol3[15] and Gaussian09.[16] For the plane-wave, pseudopotential code CASTEP, exchange and correlation were approximated using the Perdew-Burke-Ernzerhof (PBE) functional,[17] with the Tkatchenko-Scheffler (TS) dispersion correction scheme[18] within the generalized gradient approximation (GGA). The meta-GGA functional regularized Strongly Constrained and Appropriately Normed (SCAN)[19] functional (RSCAN)[20] was also used. The local density approximation (LDA) with the Ceperley and Alder functional,[21] as parameterized by Perdew and Zunger[22] (CA-PZ) with the Ortmann, Bechstedt and Schmidt[23] (OBS) van der Waals correction was also used. Norm-conserving pseudopotentials were generated by the Optimized Pseudopotential Interface / Unification Module,[24,25] or the in-built on-the-fly-generated (OTFG) pseudopotentials were used. The plane-wave cut-off and Brillouin-zone sampling of electronic states are given in Table S2. The equilibrium structure, an essential prerequisite for lattice dynamics calculations was obtained by Broyden-Fletcher-Goldfarb-Shanno (BFGS) geometry optimization after which the residual forces were converged to zero within ±0.0017 eV $Å^{-1}$. Phonon frequencies were

obtained by diagonalization of dynamical matrices computed using density-functional perturbation theory[26] (DFPT) for LDA and GGA functionals and by finite displacement for the RSCAN functional. An analysis of the resulting eigenvectors was used to map the computed modes to the corresponding irreducible representations of the point group and assign IUPAC symmetry labels. DFPT was also used to compute the dielectric response and the Born effective charges, and from these the mode oscillator strength tensor and infrared absorptivity were calculated. For DMol3, the BLYP or SCAN functional with a double numerical precision (DNP) basis set was used. For Gaussian09 the B3LYP functional with aug-ccVTZ basis set was used. The INS spectra were generated from the CASTEP output using ACLIMAX.[27]

Table S1

Refined unit cell parameters of $Fe(CO)_5$ as a function of temperature. Series 1 consists of measurements made on warming from 10 to 200 K in 10 K increments; series 2 made on cooling from 155 to 55 K in 10 K increments; series 3 made on warming from 100 to 110 K in 2 K increments and then 210 to 240 K in 10 K steps. All entries refer to the *C*-centred cell. Numbers in parentheses report the estimated standard uncertainty in the last quoted digit.

Series 1

T (K)	*a* (Å)	*b* (Å)	*c* (Å)	α (°)	β (°)	γ (°)	V (Å^3)
10	11.61136(5)	6.75987(2)	9.13832(4)	91.8032(3)	108.0216(3)	91.7703(4)	681.127(3)
20	11.61254(7)	6.75999(3)	9.14138(6)	91.7716(5)	108.0145(5)	91.7457(5)	681.495(5)
30	11.61539(8)	6.76066(3)	9.14705(7)	91.7138(5)	108.0008(5)	91.6965(5)	682.261(5)
40	11.62009(9)	6.76184(3)	9.15556(7)	91.6219(5)	107.9780(5)	91.6170(6)	683.467(5)
50	11.62533(8)	6.76281(3)	9.16293(7)	91.5374(5)	107.9617(6)	91.5424(6)	684.563(5)
60	11.63083(8)	6.76416(3)	9.17247(7)	91.4253(5)	107.9391(5)	91.4410(6)	685.921(5)
70	11.63757(8)	6.76575(3)	9.18394(7)	91.2782(5)	107.9118(5)	91.3059(6)	687.561(5)
80	11.64628(8)	6.76796(4)	9.19880(7)	91.0619(5)	107.8788(5)	91.0974(6)	689.695(5)
90	11.65377(7)	6.76976(4)	9.21235(7)	90.8260(6)	107.8524(5)	90.8620(6)	691.579(5)
100	11.66156(9)	6.77160(4)	9.22663(8)	90.4659(7)	107.8240(6)	90.4911(7)	693.561(6)
110	11.67004(12)	6.77472(6)	9.23910(13)	90	107.809(1)	90	695.451(9)
120	11.68156(9)	6.77931(5)	9.25115(9)	90	107.800(1)	90	697.555(7)
130	11.69306(9)	6.78435(4)	9.26366(9)	90	107.791(1)	90	699.739(6)
140	11.70481(8)	6.78945(4)	9.27667(8)	90	107.785(1)	90	701.977(6)
150	11.71715(8)	6.79485(4)	9.28964(8)	90	107.777(1)	90	704.290(6)
160	11.72951(8)	6.80038(4)	9.30327(7)	90	107.770(1)	90	706.670(6)
170	11.74225(8)	6.80622(4)	9.31723(7)	90	107.764(1)	90	709.134(5)
180	11.75538(7)	6.81226(4)	9.33139(7)	90	107.7581(5)	90	711.659(5)
190	11.76861(8)	6.81852(4)	9.34621(7)	90	107.751(1)	90	714.278(6)
200	11.78238(2)	6.82495(1)	9.36148(2)	90	107.744(1)	90	716.984(1)

			Series 2				
T (K)	*a* (Å)	*b* (Å)	*c* (Å)	α (°)	β (°)	γ (°)	V ($Å^3$)
155	11.72336(7)	6.79762(4)	9.296692(6)	90	107.7730(5)	90	705.503(5)
145	11.71106(7)	6.79220(4)	9.28327(7)	90	107.7810(5)	90	703.153(5)
135	11.69912(7)	6.78692(4)	9.27027(6)	90	107.7873(5)	90	700.883(5)
125	11.68738(7)	6.78179(4)	9.25759(7)	90	107.7949(5)	90	698.663(5)
115	11.67604(9)	6.77705(4)	9.24507(8)	90	107.8026(6)	90	696.523(6)
105	11.66645(14)	6.77283(6)	9.23388(13)	90.036(2)	107.806(1)	90.044(2)	694.66(1)
95	11.65769(8)	6.77063(4)	9.21954(7)	90.6644(6)	107.8355(6)	90.6990(6)	692.582(6)
85	11.64991(8)	6.76879(3)	9.20564(7)	90.9481(5)	107.8637(5)	90.9879(6)	690.636(5)
75	11.64241(8)	6.76704(3)	9.19249(7)	91.1571(5)	107.8927(5)	91.1920(6)	688.780(5)
65	11.63402(8)	6.76493(3)	9.17820(7)	91.3519(5)	107.9235(5)	91.3773(6)	686.734(5)
55	11.62797(8)	6.76342(3)	9.16713(7)	91.4868(5)	107.9508(5)	91.5013(5)	685.179(5)

			Series 3				
T (K)	*a* (Å)	*b* (Å)	*c* (Å)	α (°)	β (°)	γ (°)	V ($Å^3$)
100	11.66166(6)	6.77160(3)	9.22661(6)	90.4552(5)	107.8226(4)	90.4807(5)	693.574(4)
102	11.66313(10)	6.77203(4)	9.22964(9)	90.3350(8)	107.8172(7)	90.3530(8)	693.986(7)
104	11.66506(16)	6.77260(7)	9.23254(19)	90.135(1)	107.810(1)	90.149(1)	694.43(1)
106	11.66571(11)	6.77282(5)	9.23422(10)	90	107.8103(8)	90	694.627(7)
108	11.66826(10)	6.77377(5)	9.23653(9)	90	107.8088(7)	90	695.056(7)
110	11.67038(6)	6.77468(3)	9.23889(6)	90	107.8061(4)	90	695.464(4)
210	11.79618(8)	6.83170(4)	9.37700(7)	90	107.7371(5)	90	719.753(5)
220	11.81031(8)	6.83867(4)	9.39299(7)	90	107.729(1)	90	722.613(6)
230	11.82489(9)	6.84605(4)	9.40977(8)	90	107.723(1)	90	725.601(6)
240	11.83970(9)	6.85365(4)	9.42682(8)	90	107.716(1)	90	728.663(6)

Table S2

Parameters resulting from fitting polynomials of the form $X = X_0 + yT + zT^2$ to the unit-cell parameters of the $C2/c$ phase of $Fe(CO)_5$.

	X_0	y	z
a-axis	11.5657(5)	7.90(6) x10^{-4}	1.47(2) x10^{-6}
b-axis	6.7388(4)	1.99(5) x10^{-4}	1.16(1) x10^{-6}
c-axis	9.1336(11)	7.41(13) x10^{-4}	2.00(4) x10^{-6}
Angle β	107.882(1)	−6.94(6) x10^{-4}	–

Figure S1

Neutron powder diffraction pattern of $Fe(CO)_5$ measured at 10 K on the HRPD instrument at ISIS. The observations are plotted as filled red circles, the fitted profile refinement as a solid green line, and the difference profile as a solid purple line underneath the Bragg peak markers. The latter are indicated by vertical black lines.

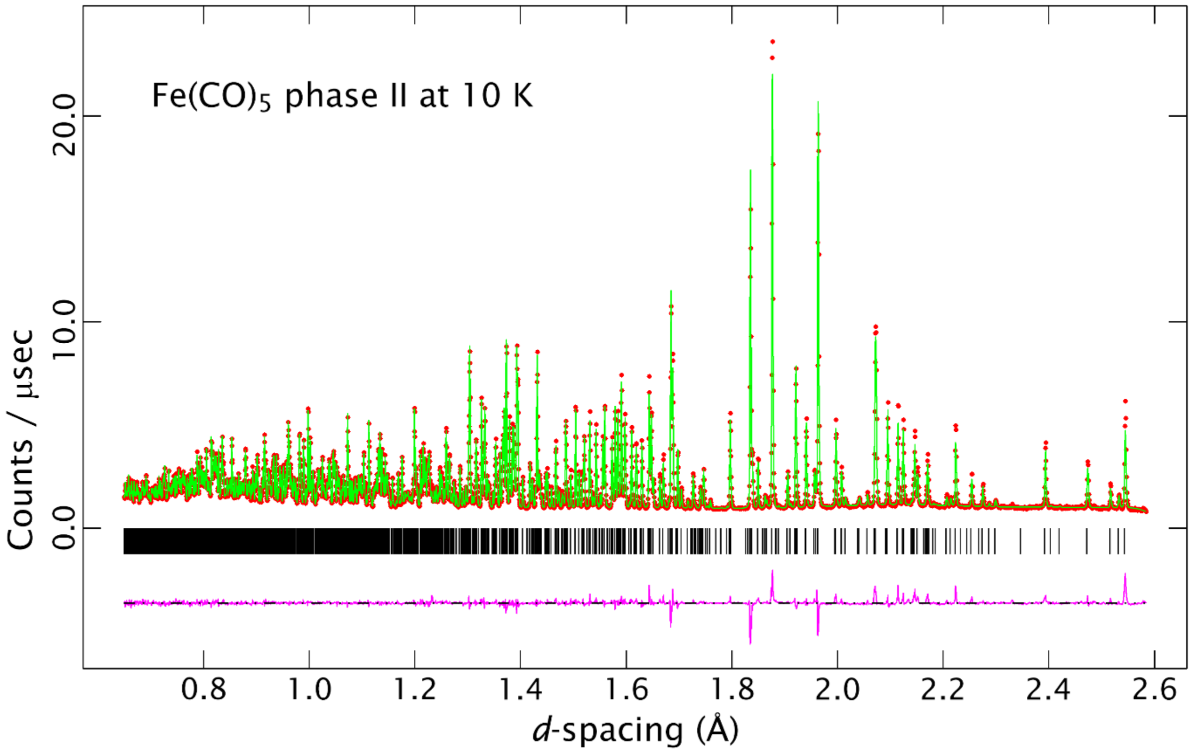

Figure S2

Neutron powder diffraction pattern of $Fe(CO)_5$ measured at 200 K on the HRPD instrument at ISIS. The observations are plotted as filled red circles, the fitted profile refinement as a solid green line, and the difference profile as a solid purple line underneath the Bragg peak markers. The latter are indicated by vertical black lines.

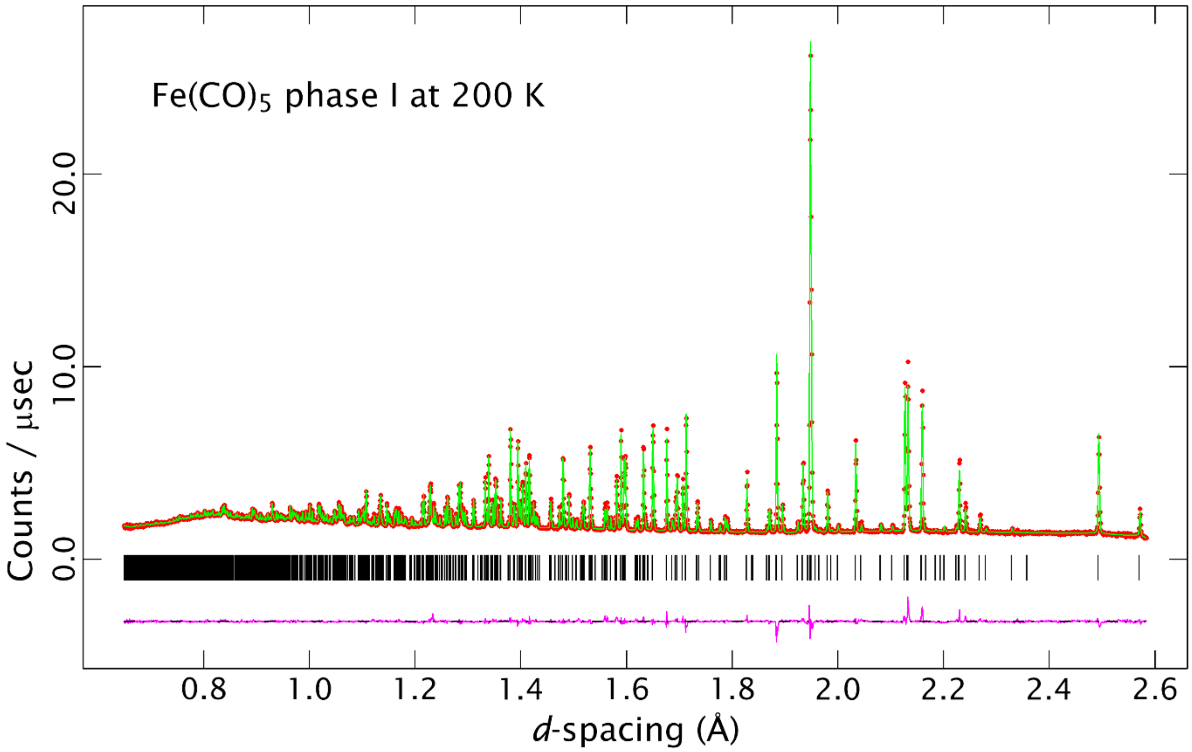

Figure S3

Elements, e_{ij}, of the spontaneous strain tensor as a function of temperature, corresponding with the strain resulting from the ferroelastic transition from the $C2/c$ to $C\bar{1}$ structure on cooling of $Fe(CO)_5$. The panel on the left emphasises the large symmetry-breaking strains, e_{12} and e_{23}. The panel on the right offers an expanded view to highlight the linear dependence on T of the weak non symmetry-breaking strains.

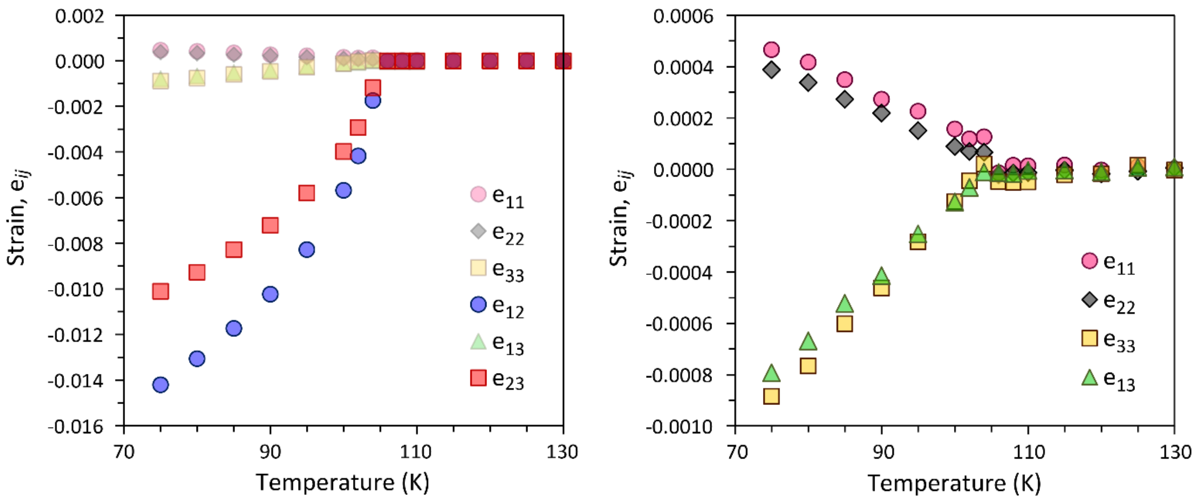

Figure S4

Representation surface of the spontaneous strain tensor, evaluated at 75 K. The lobes coloured in red indicate negative tensile strain along the principal direction e_1; the lobes in green indicate positive tensile strain along the principal direction e_3. The orthogonal axes are related to the crystallographic axes of the *C*-centred cell by, x || *a**, y || *b*, z || *c*. The spatial relationship of this figure to the crystal structure is shown in the main text, Figure 5. Drawn using *WinTensor*[28]

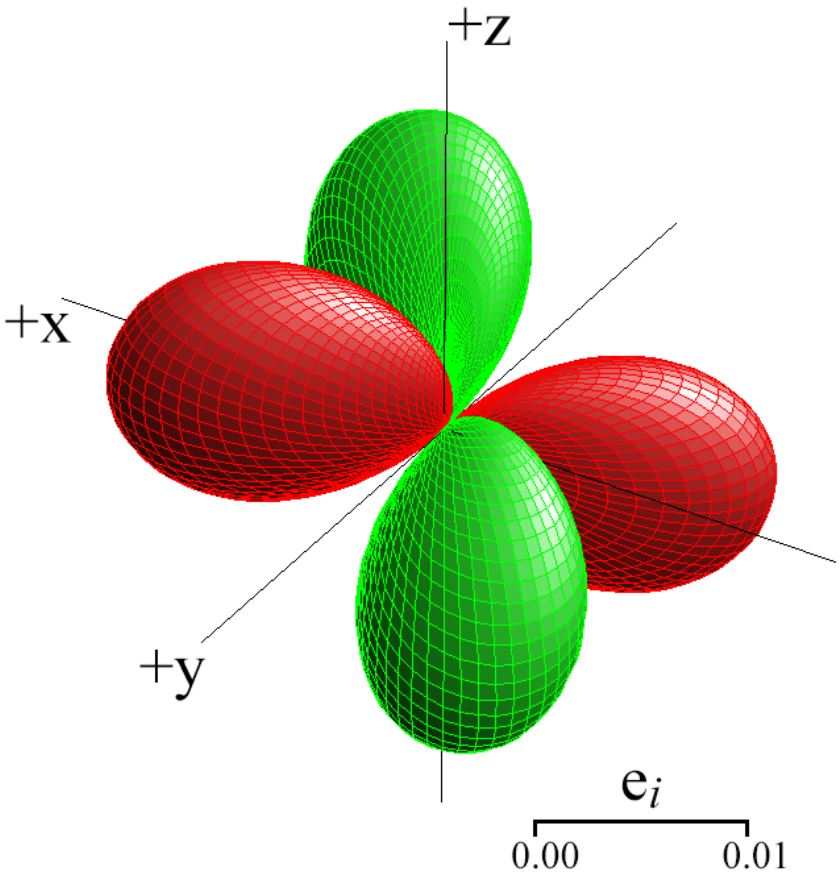

Figure S5

Another proxy for the spontaneous strain element e_{23}, that may be calculated without recourse to extrapolation of the high-temperature unit-cell parameters, is $\cos^2(\alpha^*)$, where the * denotes the reciprocal lattice. The panel on the left shows the linear relationship between $\cos^2(\alpha^*)$ and temperature, with an intercept at $T_C = 104.6(3)$ K. The panel on the right shows the perfectly linear relationship between $\cos(\gamma)$ and $\cos(\alpha^*)$ in the range of temperatures from 70 – 100 K, indicating that a single order parameter, Q, is responsible for both the e_{12} and e_{23} strains with comparable degrees of coupling.

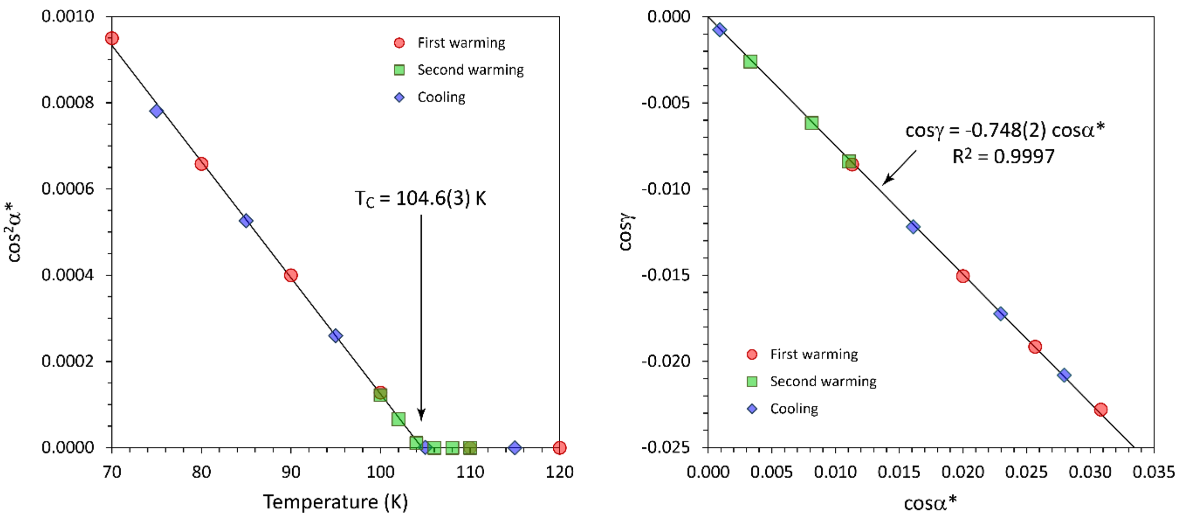

Figure S6

Fingerprint plots derived from calculated Hirschfeld surfaces. The contributions from different intermolecular contacts are shown as a function of temperature; these are the C···O contacts and their reciprocal O···C contacts, O···O contacts and C···C contacts. For each panel, the fractional contribution to the total surface area of the Hirschfeld surface is reported.

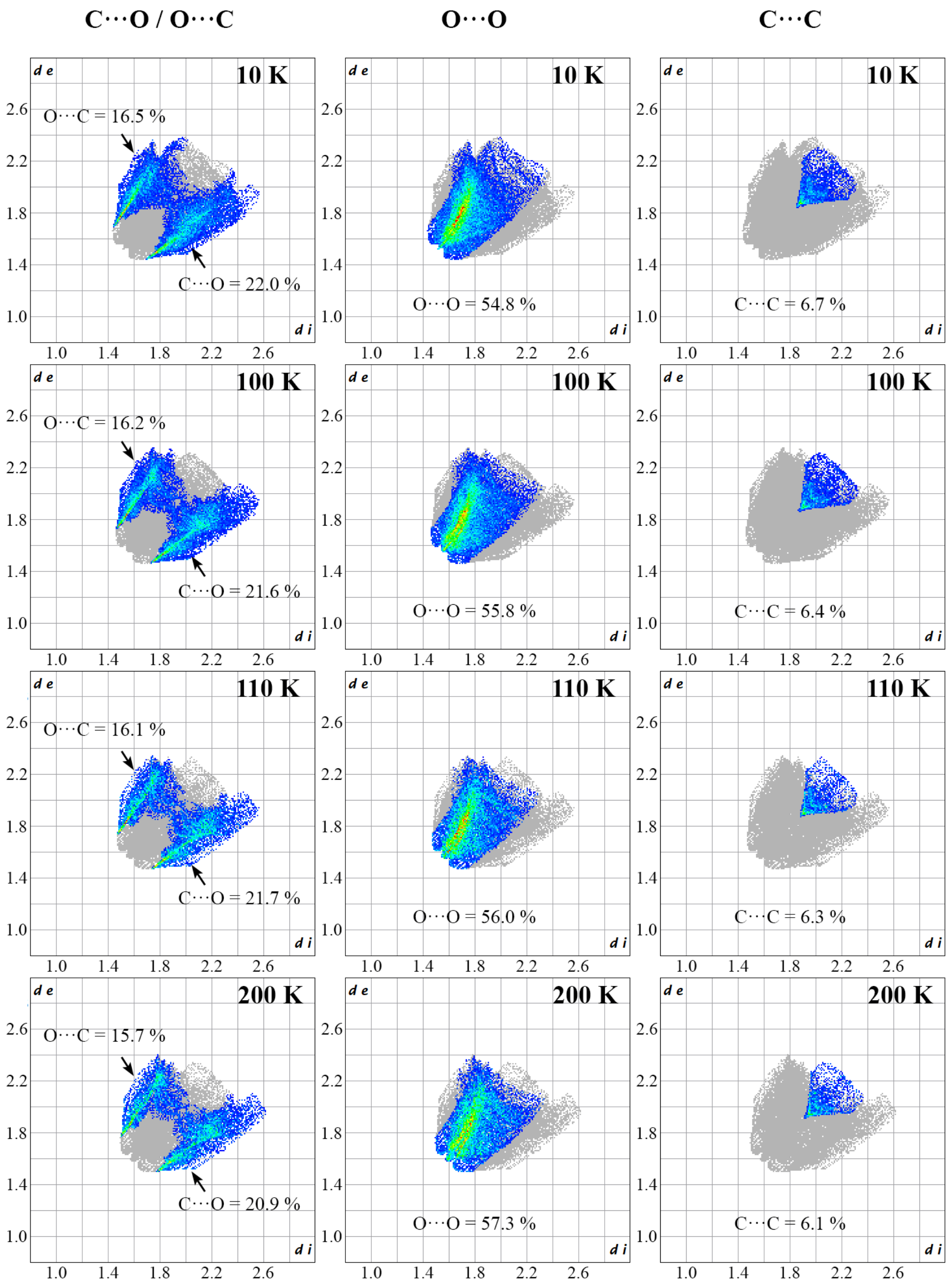

Figure S7

Hirschfeld surfaces of the $Fe(CO)_5$ molecule at temperatures corresponding with the four structural refinements reported in this work. Surfaces are shaded by d_{norm} values, with white and blue corresponding to distances that are equal to, or longer than, the sum of the van der Waals radii. Patches of red colour correspond with distances shorter than the van der Waals radii sum.

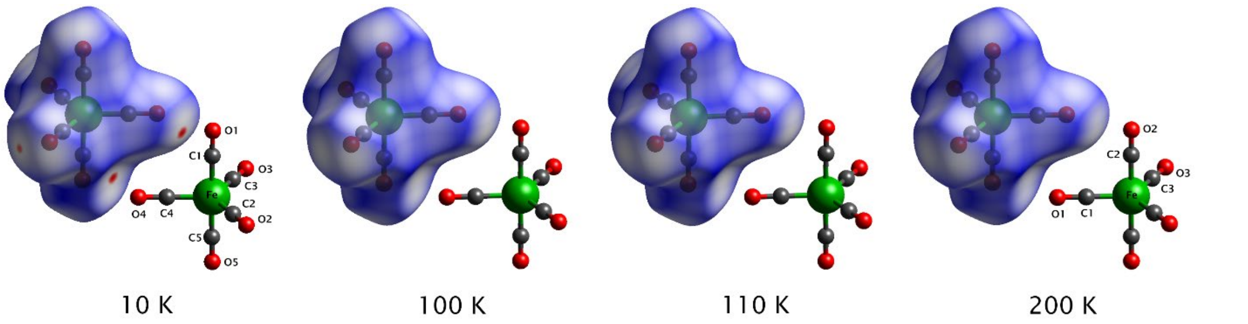

Figure S8

The three strongest symmetry inequivalent interactions between near-neighbour $Fe(CO)_5$ molecules (a) and the three weakest such interactions (b). The non color-coded molecule labelled '0' is at coordinates *x*,*y*,*z*: the color-coded molecules illustrated are sited at (1) 1−*x*,−*y*,2−*z*; (2) ½−*x*, ½−*y*,1−*z*; (3) 1−*x*,1−*y*,1−*z*; (4) *x*−½,*y*−½,*z*; (5) *x*,1+*y*,*z*; (6) $^3/_2$−*x*, ½−*y*,1−*z*. The spatial relationships of the interactions are as follows: 0–1 has the bisectors of the O1–C1, O3–C3 carbonyls parallel and vertically offset; 0–2 has the O1–C1 carbonyls parallel with a small lateral offset; 0–3 has the O1–C1 carbonyls parallel with a larger lateral offset; 0–4 has the axial carbonyls parallel; 0–5 has the O3–C3 carbonyl parallel to the O1–C1 and O3–C3 bisector, pointing directly at the Fe atom; 0–6 has the axial carbonyls parallel but with a larger lateral and vertical offset.

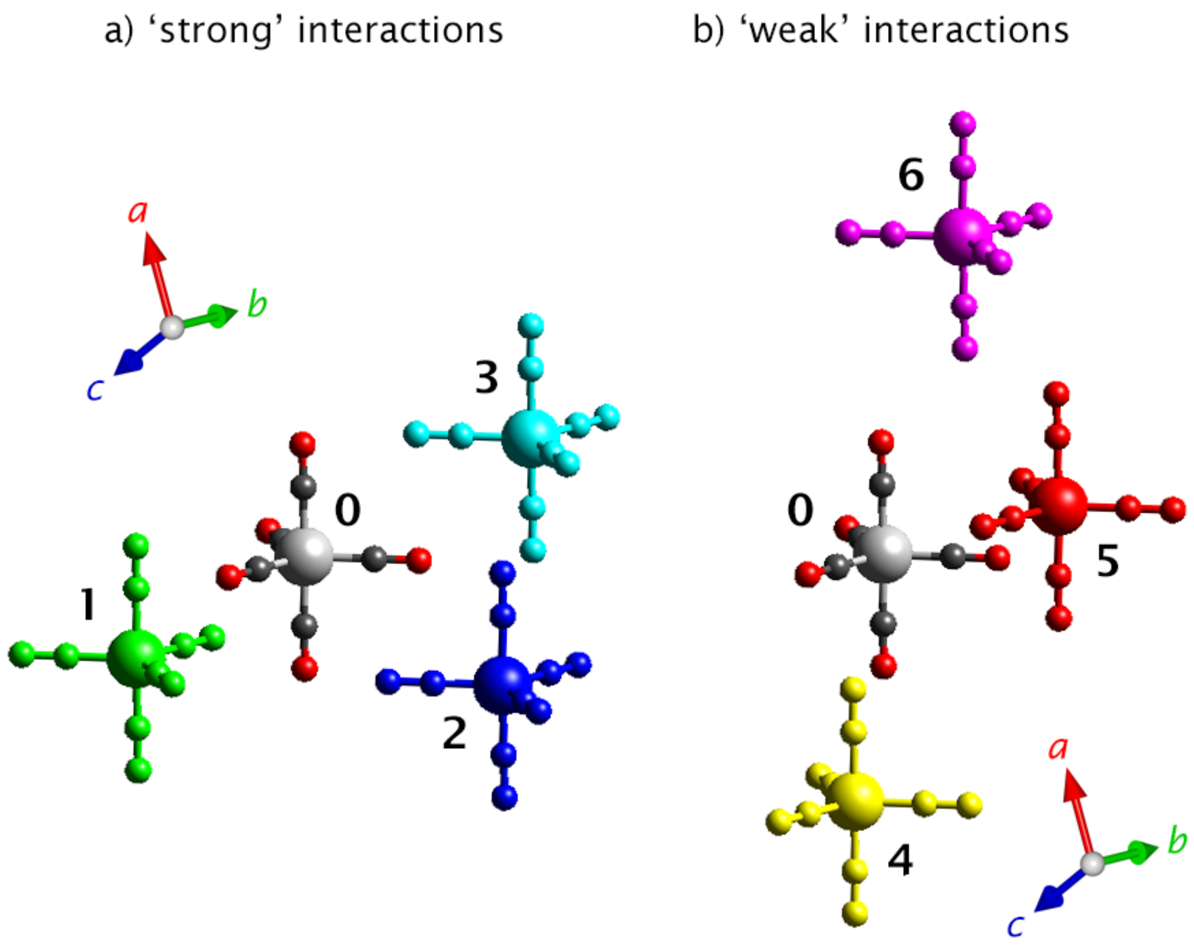

Table S3

Energies associated with each of the interactions depicted in Figure S8, evaluated at 200 K, using the same colour-coding scheme. The energies are broken down into electrostatic (E_{ele}), polarisation (E_{pol}), dispersion (E_{dis}) and exchange-repulsion (E_{rep}) contributions. The total energy of each interaction is E_{tot}. R is the distance between molecular centres.

			Interaction energies (kJ mol^{-1})				
Interaction		R (Å)	E_{ele}	E_{pol}	E_{dis}	E_{rep}	E_{tot}
0–1		5.21	−2.1	−0.6	−16.8	9.9	−11.2
0–2		6.41	−5.0	−0.5	−10.2	7.9	−9.7
0–3		6.52	−3.9	−0.4	−8.7	4.8	−9.0
0–4		6.81	−0.2	−0.1	−6.3	2.2	−4.0
0–5		6.82	−2.5	−0.2	−6.3	6.3	−4.4
0–6		8.64	−0.1	−0.0	−1.8	0.4	−1.5

Table S4

Temperature dependence of the interaction energies for the first nearest neighbour interaction, 0–1.

**0–1**		Interaction energies (kJ mol^{-1})					Ratio
T (K)	R (Å)	E_{ele}	E_{pol}	E_{dis}	E_{rep}	E_{tot}	E_{rep} / E_{dis}
200	5.21	−2.1	−0.6	−16.8	9.9	−11.2	0.5893
110	5.14	−2.6	−0.7	−18.2	12.0	−11.7	0.6593
100	5.15	−2.7	−0.7	−18.2	12.1	−11.8	0.6648
10	5.13	−2.8	−0.7	−19.1	13.1	−12.0	0.6859

Table S5

Temperature dependence of the interaction energies for the second nearest neighbour interaction, 0–2. Note the substantially larger value of the ratio E_{rep} / E_{dis} for this interaction compared with the other two strong stabilising interactions in the crystal (*cf.*, Tables S4 and S6).

**0–2**		Interaction energies (kJ mol^{-1})					Ratio
T (K)	R (Å)	E_{ele}	E_{pol}	E_{dis}	E_{rep}	E_{tot}	E_{rep} / E_{dis}
200	6.41	−5.0	−0.5	−10.2	7.9	−9.7	0.7745
110	6.34	−5.1	−0.5	−10.9	9.4	−9.5	0.8624
100	6.30	−5.0	−0.5	−11.0	9.4	−9.4	0.8545
10	6.16	−5.2	−0.5	−12.0	11.2	−9.3	0.9333

Table S6

Temperature dependence of the interaction energies for the third nearest neighbour interaction, 0–3.

**0–3**		Interaction energies (kJ mol^{-1})					Ratio
T (K)	R (Å)	E_{ele}	E_{pol}	E_{dis}	E_{rep}	E_{tot}	E_{rep} / E_{dis}
200	6.52	−3.9	−0.4	−8.7	4.8	−9.0	0.5517
110	6.46	−4.1	−0.4	−9.4	5.9	−9.1	0.6277
100	6.42	−4.3	−0.4	−9.8	6.4	−9.4	0.6531
10	6.27	−4.9	−0.5	−11.0	8.6	−9.8	0.7818

Thermal expansion analysis

Eulerian infinitesimal strain tensors were calculated from pairs of cell parameters determined at adjacent temperature and then normalised by the temperature increment between them in order to obtain thermal expansion tensors, *i.e.*, unit-strain tensors (*cf.*[29]). Standard matrix decomposition methods[30] were used to derive the eigenvalues and eigenvectors of the thermal expansion tensor, these being the magnitudes and orientations of the principal expansivities.

The temperature dependences of the three principal linear expansivities are shown in Figure S8, revealing the involvement of the spontaneous strain in dominating the linear thermal expansion terms α_1 and α_2. Above the transition, in the absence of the spontaneous strain, we see that the underlying framework expansivity is only moderately anisotropic (Figure S8d).

Figure S9

Principal thermal expansivities as a function of temperature: (a) α_1; (b) α_2; (c) α_3; (d) plot of α_1–α_3 above the ferroelastic transition on the same scale.

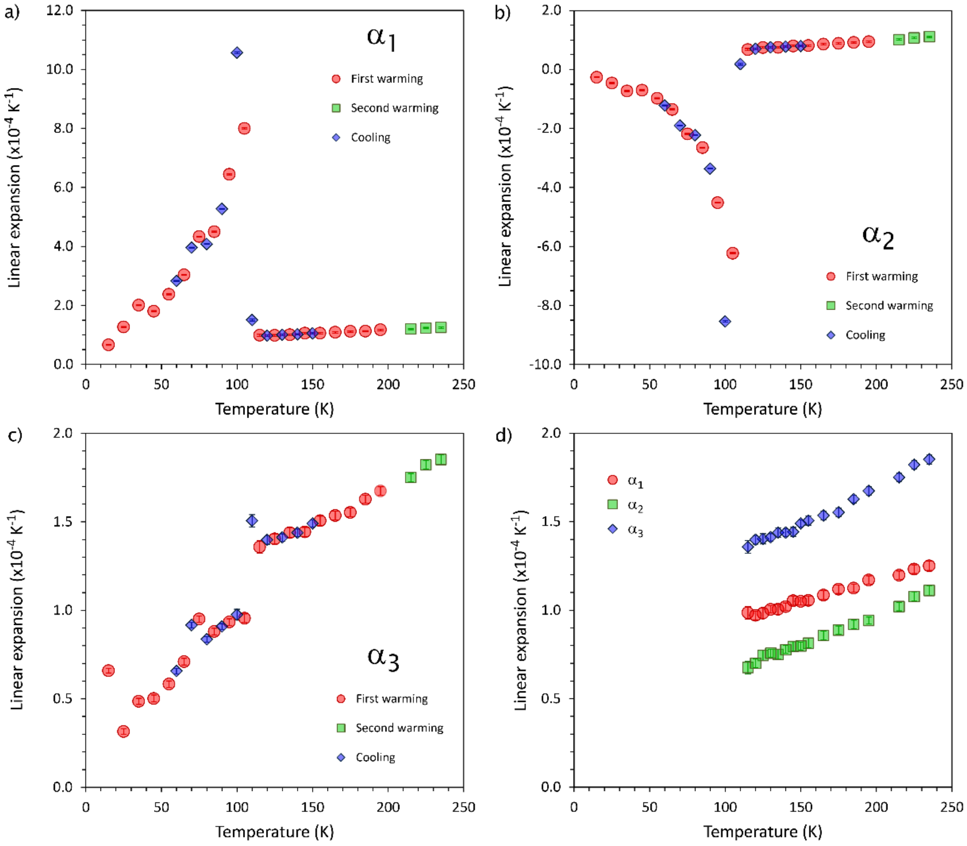

The principal direction α_2 for the monoclinic phase of $Fe(CO)_5$ is constrained by symmetry to coincide with the 2-fold axis, whilst α_1 and α_3 need not be aligned with any particular crystallographic direction; in fact, the direction of greatest thermal expansion above 110 K, α_3, is approximately parallel with *c**. In the low-temperature triclinic phase of $Fe(CO)_5$ the orientation of the thermal expansion tensor has no such symmetry constraints and the principal directions do not necessarily co-align with any particular direction. However, α_1 and α_2 *are* co-planar with the spontaneous strains e_1 and e_3, lying approximately in the $(\bar{1}01)$ plane. Figure S9 shows the spatial relationship between the thermal expansion tensor representations (*cf.*[31]) above and below the ferroelastic transition and the spontaneous strain tensor representation below the transition.

Figure S10

Comparison of the tensor representation surfaces describing (a) the spontaneous strain at 75 K, (b) the thermal expansion at 95 K, and (c) the thermal expansion at 120 K. The green lobes indicate positive strain and the red lobes denote negative strain. The orthogonal axes are related to the crystallographic axes of the *C*-centred cell by, x || *a**, y || *b*, z || *c*. Drawn using *WinTensor*.[28]

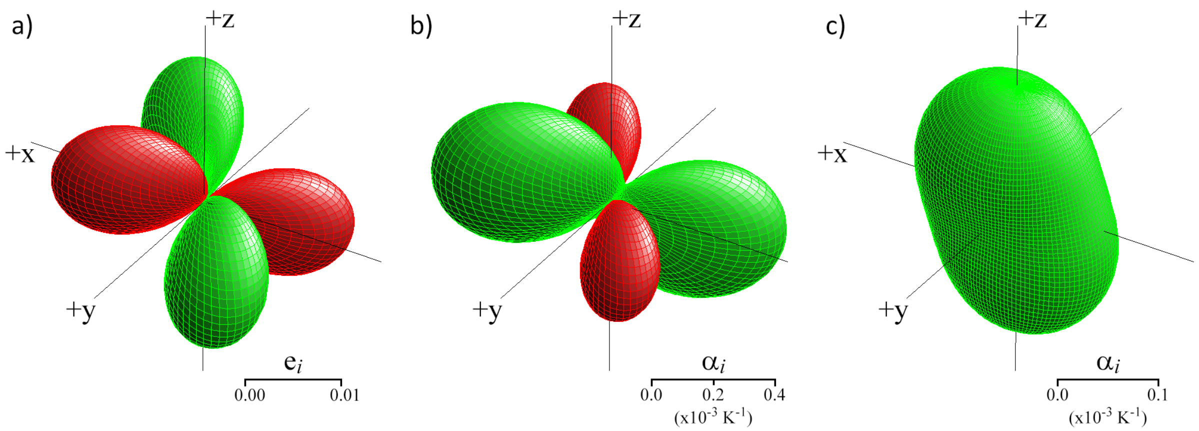

The temperature dependence of the volume thermal expansion is plotted in Figure S10. The large anomalies present in the principal expansivities largely cancel out and the volume expansion is a smoothly varying function from 10 – 240 K, as one might expect for a second-order phase transition. This agrees with the lack of any observed anomaly in the specific heat capacity.[32]

Figure S11

Volume thermal expansion of $Fe(CO)_5$.

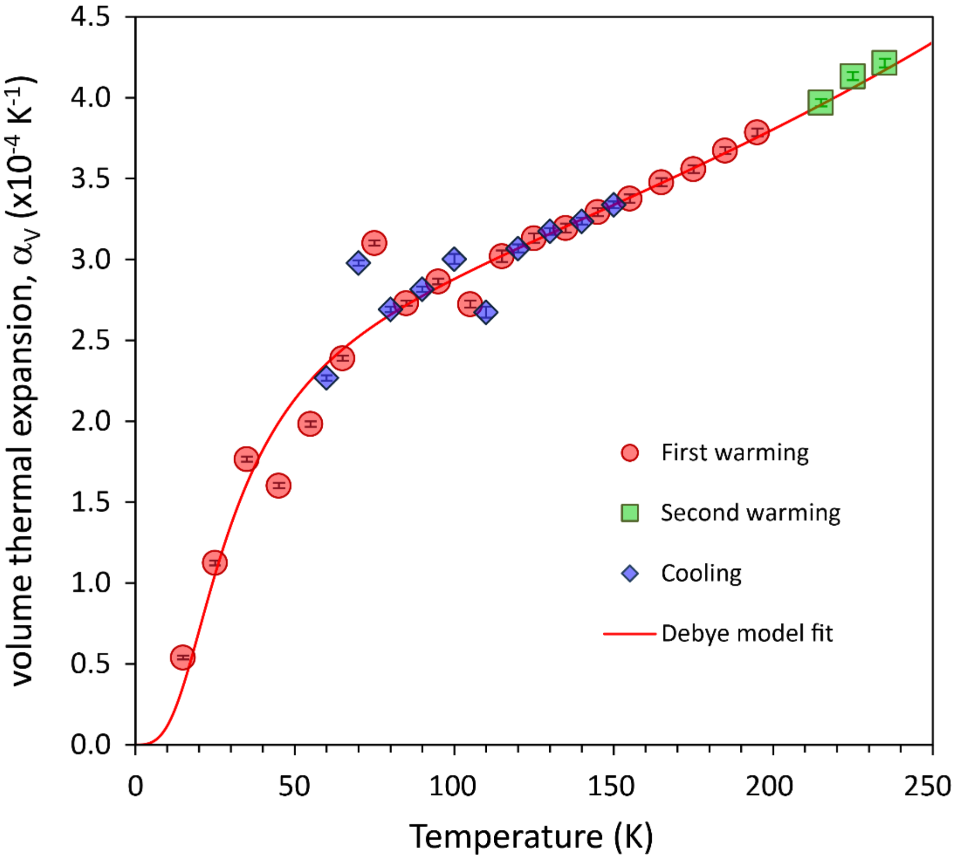

The unit-cell volumes of $Fe(CO)_5$, in both the low- and high-temperature forms, have been fitted with a second-order Grüneisen approximation to the zero-pressure equation of state.[33] In this approximation, the thermal expansion is considered equivalent to elastic strain such that,

$$V(T) = V_0\left[1 + \frac{E(T)}{Q - bE(T)}\right] \tag{1}$$

where V_0 is the unit cell volume at zero pressure, $b = ½\,(K_0'-1)$ and $Q = (V_0\,K_0/\gamma)$; K_0 is the zero-pressure isothermal bulk modulus, K_0' is its first derivative with respect to pressure, and γ is the thermal Grüneisen parameter. The internal energy due to lattice vibrations, $E(T)$, is then determined via a simple Debye model approximation of the phonon density of states:

$$E(T) = \frac{9nk_BT}{(\theta_D/T)^3} \int_0^{\theta_D/T} \frac{x^3}{e^x - 1} dx \qquad (2)$$

where θ_D is the Debye temperature, n is the number of atoms per unit cell, and k_B is the Boltzmann constant; the integral term is evaluated numerically.

Table S4 reports the parameters obtained from fitting equations 1 to the unit-cell volumes of $Fe(CO)_5$. These correspond with the solid red line on Figure S11.

Table S7

Lines 1 to 4 report parameters obtained by the fitting of a Debye-type model to the unit-cell volume of $Fe(CO)_5$ as a function of temperature (Eq. 1 and 2). The last four rows of the table report values derived from the parameters in the preceding rows.

	Unit cell volume
θ_D (K)	122(3)
V_0 (cm^3 mol^{-1})	102.555(9)
Φ (J cm^{-3})	1.01(1)x10^6
b	4.2(1)
Debye cut-off (cm^{-1})	85(2)
V_0 (Å^3)	681.18(6)
K_0/γ (GPa)	9.8(1)
K'_0	9.4(3)

Table S8

Bond lengths and angles of $Fe(CO)_5$ as a function of temperature.

	$C2/c$		$P\bar{1}$	
	200 K	110 K	100 K	10 K
Bond lengths (Å)				
C1–O1	1.156(2)	1.147(2)	1.146(3)	1.147(2)
C4–O4			1.148(3)	1.147(2)
C2–O2	1.111(1)	1.122(2)	1.119(3)	1.124(2)
C5–O5			1.118(3)	1.125(2)
C3–O3	1.131(2)	1.130(2)	1.129(3)	1.138(2)
Fe1–C1	1.809(2)	1.807(2)	1.809(3)	1.814(1)
Fe1–C4			1.805(3)	1.812(2)
Fe1–C2	1.824(1)	1.823(1)	1.826(3)	1.824(2)
Fe1–C5			1.824(3)	1.829(2)
Fe1–C3	1.814(2)	1.816(2)	1.816(3)	1.816(1)
Bond angles (°)				
C1–Fe1–C1(i)	117.7(1)	117.3(1)		
C1–Fe1–C4			116.8(1)	116.41(7)
C1–Fe1–C3	121.18(5)	121.38(5)	121.5(1)	120.20(7)
C4–Fe1–C3			121.8(1)	123.39(7)
C2–Fe1–C1	90.51(5)	90.36(6)	90.6(1)	90.64(7)
C2–Fe1–C1(i)	90.20(6)	90.23(6)		
C2–Fe1–C4			90.3(1)	89.93(7)
C2–Fe1–C3	89.32(6)	89.43(6)	89.5(1)	89.55(6)
C2–Fe1–C2(i)	178.6(1)	178.9(1)		
C2–Fe1–C5			178.8(1)	178.45(8)
Fe1–C1–O1	179.6(1)	179.8(2)	179.4(2)	179.8(1)
Fe1–C4–O4			179.5(2)	179.6(1)
Fe1–C2–O2	179.8(1)	179.3(2)	179.1(2)	179.4(1)
Fe1–C5–O5			179.6(2)	179.3(2)
Fe1–C3–O3	180.0	180.0	179.3(3)	179.2(2)

(i) 1−x, y, 1½−z

Table S9

Comparison of CASTEP[a] calculated geometry of $Fe(CO)_5$ with the experimental structure.

Functional[b]	Expt.	Std<br>PBE w/ TS	Std_v2<br>PBE w/ TS	Opium<br>PBE w/ TS	LDA<br>CA-PZ w/ OBS	CASTEP20<br>PBE w/ TS	CASTEP20<br>RSCAN
Type[c]		GGA	GGA	GGA	LDA	GGA	meta-GGA
Plane wave cut-off / eV		1440	1440	990	1440	1440	1440
Pseudopotentials[d]		OTFG norm conserving	OTFG norm conserving	Opium	OTFG norm conserving	OTFG norm conserving	OTFG norm conserving
k-point grid		8 × 8 × 5	9 × 8 × 6	9 × 8 × 6	9 × 8 × 6	8 × 8 × 5	8 × 8 × 5
Number of k-points		160	216	216	216	160	160
Distance / Å							
Fe–C_{ax}	1.829, 1.824	1.791, 1.793	1.791, 1.793	1.775, 1.776	1.768, 1.770	1.791, 1.793	1.786, 1.787
Fe–C_{eq}	1.816, 1.814, 1.813	1.786, 1.786, 1.784	1.786, 1.786, 1.784	1.765, 1.762, 1.765	1.763, 1.760, 1.762	1.786, 1.786, 1.784	1.776, 1.778, 1.778
C≡O_{ax}	1.124, 1.125	1.154, 1.154	1.154, 1.154	1.144, 1.144	1.148, 1.148	1.154, 1.154	1.134, 1.134
C≡O_{eq}	1.138, 1.147, 1.147	1.158. 1.158, 1.158	1.158. 1.158, 1.158	1.148, 1.149, 1.149	1.152, 1.153, 1.152	1.158. 1.158, 1.158	1.139. 1.139, 1.139
Angle / °							
C_{ax}-Fe-C_{ax}	178.4	178.2	178.2	178.2	178.5	178.2	178.4
C_{ax}-Fe-C_{eq}	89.0, 89.9, 89.6	89.1, 90.6, 90.3	89.1, 90.5, 90.3	89.1, 90.6, 90.2	89.0, 91.6, 90.0	89.1, 90.6, 90.3	89.7, 90.5, 90.1
C_{eq}-Fe-C_{eq}	123.3, 120.2, 116.4	123.7, 119.8, 116.4	123.7, 119.9, 116.3	123.5, 1119.9, 116.6	122.1, 120.8, 117.1	123.7, 119.8, 116.4	122.8, 120.1, 117.1

[a]All calculations started from our experimentally determined 10 K neutron diffraction structure. Columns 2 - 4 used CASTEP v17, the last two columns used CASTEP v20.[14]
[b]PBE w/ TS is the Perdew-Becke-Ernzerhof[17] functional with the Tkatchenko and Scheffler[18] van der Waals correction. CA-PZ w/ OBS is the Ceperley and Alder functional[21] as parameterized by Perdew and Zunger[22] with the Ortmann, Bechstedt and Schmidt[23] van der Waals correction. RSCAN[20] is the regularized Strongly Constrained and Appropriately Normed (SCAN)[19] functional.
[c]GGA = generalized gradient approximation. LDA = local gradient approximation. meta-GGA = meta generalized gradient approximation.
[d]OTFG = on-the-fly-generated pseudopotential. Opium = norm-conserving pseudopotentials generated by the Optimized Pseudopotential Interface / Unification Module.[24,25]

Additional Supplementary Information

Crystallographic Information Files (CIFs) reporting the atomic coordinates and anisotropic displacement parameters at 10, 100, 110 and 200 K. These include additional quantitative information about the measurements and the refinements, and also contain the observed and calculated neutron powder diffraction patterns.

These files are deposited with the Cambridge Structural Database and may be accessed using the following codes.

$Fe(CO)_5$ at 10 K: CSD 2126350

$Fe(CO)_5$ at 100 K: CSD 2126351

$Fe(CO)_5$ at 110 K: CSD 2126352

$Fe(CO)_5$ at 200 K: CSD 2126353

References

[1] Ibberson, R. M.; David W. I. F.; Knight, K. S. The high resolution neutron powder diffractometer (HRPD) at ISIS – a user guide. RAL-92-031. Rutherford Appleton Laboratory, U.K., 1992. Available online: http://purl.org/net/epubs/work/25156

[2] Ibberson, R. M. Design and performance of the new supermirror guide on HRPD at ISIS. *Nucl. Instr. Methods Phys. Res. A*. **2009**, *600*, 47-49.

[3] https://www.isis.stfc.ac.uk/Pages/About.aspx

[4] Fortes, A. D. Accurate and precise lattice parameters of H_2O and D_2O ice I*h* between 1.6 and 270 K from high-resolution time-of-flight neutron powder diffraction data. *Acta Crystallogr. Sect. B*. **2018**, **74**, 196-216.

[5] Fortes, A. D.; Capelli, S. C. H/D isotope effect on the molar volume and thermal expansion of benzene. *Phys. Chem. Chem. Phys*. **2018**, *20*, 16736-16742.

[6] Arnold, O. and 27 co-authors. Mantid—Data analysis and visualization package for neutron scattering and μSR experiments *Nucl. Instrum. Methods Phys. Res. A*. **2014**, *764*, 156-166.

[7] MANTID (2013) Manipulation and Analysis Toolkit for Instrument Data; Mantid Project. http://dx.doi.org/10.5286/SOFTWARE/MANTID.

[8] Larson, A. C.; Von Dreele, R. B. *General Structure Analysis System (GSAS)*, Los Alamos National Laboratory Report, LAUR 86-748, 1994.

[9] Toby, B. H. *EXPGUI*, a graphical user interface for *GSAS*. *J. Appl. Cryst*. **2001**, *34*, 210-213.

[10] R. Boese and D. Bläser, Crystal structure refinement of pentacarbonyliron, $Fe(CO)_5$. *Z. Kristallogr.* **1990**, *193*, 289-290.

[11] Parker, S. F.; Fernandez-Alonso, F.; Ramirez-Cuesta, A. J.; Tomkinson, J.; Rudić, S.; Pinna, R. S.; Gorini, G.; Fernández Castañon, J. Recent and future developments on TOSCA at ISIS. *J. Phys. Conf. Ser.* **2014**, *554*, 012003.

[12] Pinna, R. S.; Rudić, S.; Parker, S. F.; Armstrong, J.; Zanetti, M.; Škoro, G.; Waller, S. P.; Zacek, D.; Smith, C. A.; Capstick, M. J.; McPhail, D. J.; Pooley, D. E.; Howells, G. D.; Gorini, G.; Fernandez-Alonso, F. The neutron guide upgrade of the TOSCA spectrometer. *Nucl. Inst. Meth. Phys. Res. Section A* **2018**, *896*, 68–74.

[13] Adams, M. A.; Parker, S. F.; Fernandez-Alonso, F.; Cutler, D. J.; Hodges, C.; King, A. Simultaneous neutron scattering and Raman scattering. *Appl. Spec.* **2009**, *63*, 727-732.

[14] Clark, S. J.; Segall, M. D.; Pickard, C. J.; Hasnip, P. J.; Probert, M. J.; Refson, K.; Payne, M. C. First principles methods using CASTEP. *Z. Krist*. **2005**, *220*, 567.

[15] Delley, B. From molecules to solids with the DMol^3 approach. *J. Chem. Phys*. **2000**, *113*, 7756-7764.

[16] Gaussian 09, Frisch, M. J.; Trucks, G. W.; Schlegel, H. B.; Scuseria, G. E.; Robb, M. A.; Cheeseman, J. R.; Scalmani, G.; Barone, V.; Petersson, G. A.; Nakatsuji, H.; Li, X.; Caricato, M.; Marenich, A.; Bloino, J.; Janesko, B. G.; Gomperts, R.; Mennucci, B.; Hratchian, H. P.; Ortiz, J. V.; Izmaylov, A. F. Sonnenberg, J. L. Williams-Young, D. Ding, F. Lipparini, F. Egidi, F. Goings, J. Peng, B.; Petrone, A.; Henderson, T.; Ranasinghe, D.; Zakrzewski, V. G.; Gao, J.; Rega, N.; Zheng, G.; Liang, W.; Hada, M.; Ehara, M.; Toyota, K.; Fukuda, R.; Hasegawa, J.; Ishida, M.; Nakajima, T.; Honda, Y.; Kitao, O.; Nakai, H.; Vreven, T.; Throssell, K.; Montgomery, Jr.; J. A.; Peralta, J. E.; Ogliaro, F.; Bearpark, M.; Heyd, J. J.; Brothers, E.; Kudin, K. N.; Staroverov, V. N.; Keith, T.; Kobayashi, R.; Normand, J.; Raghavachari, K.; Rendell, A.; Burant, J. C.; Iyengar, S. S.; Tomasi, J.; Cossi, M.; Millam, J. M.; Klene, M.; Adamo, C.; Cammi, R.; Ochterski, J. W.; Martin, R. L.; Morokuma, K.; Farkas, O.; Foresman, J. B.; Fox, D. J. Gaussian, Inc., Wallingford CT, 2016.

[17] Perdew, J.; Burke, K.; Ernzerhof, M. Generalized gradient approximation made simple. *Phys. Rev. Lett*. **1996**, *77*, 3865.

[18] Tkatchenko. A.; Scheffler, M. Accurate molecular van der Waals interactions from ground-state electron density and free-atom reference data. *Phys. Rev. Lett*. **2009**, *102*, 073005.

[19] Sun, J.; Remsing, R. C.; Zhang, Y.; Sun, Z.; Ruzsinszky, A.; Peng, H.; Yang, Z.; Paul, A.; Waghmare, U.; Wu, X.; Klein M. L.; Perdew, J. P. Accurate first-principles structures and energies of diversely bonded systems from an efficient density functional. *Nat. Chem.* **2016**, *8*, 831-836.

[20] Bartók, A. P.; Yates, J. R. Regularized SCAN functional. *J. Chem. Phys.* **2019**, *150*, 161101.

[21] Ceperley, D. M.; Alder, B. J. Ground state of the electron gas by a stochastic method. *Phys. Rev. Lett.* **1980**, *45*, 566-569.

[22] Perdew J. P.; Zunger, A. Self-interaction correction to density-functional approximations for many-electron systems. *Phys. Rev. B* **1981**, *23*, 5048-5079.

[23] Ortmann, F.; Bechstedt F.; Schmidt, W. G. Semiempirical van der Waals correction to the density functional description of solids and molecular structures. *Phys. Rev. B* **2006**, *73*, 205101.

[24] Rappe, A. M.; Rabe, K. M.; Kaxiras E.; Joannopoulos, J. D. Optimized pseudopotentials. *Phys. Rev. B* **1990**, *41*, 1227.

[25] Opium - pseudopotential generation project. http://opium.sourceforge.net/index.html

[26] Refson, K.; Clark, S. J.; Tulip, P. R.; Variational density-functional perturbation theory for dielectrics and lattice dynamics. *Phys. Rev. B* **2006**, *73*, 155114.

[27] Ramirez-Cuesta, A. J. aCLIMAX 4.0.1, The new version of the software for analyzing and interpreting INS spectra. *Comp. Phys. Comm*. **2004**, *157,* 226-238.

[28] W. Kaminski (2004) *WinTensor* 1.1 (http://cad4.cpac.washington.edu/WinTensorhome/WinTensor.htm)

[29] R. M. Hazen, R. T. Downs and C. T. Prewitt, Principles of comparative crystal chemistry. *Rev. Mineral. Geochem*. 2000, **41**, 1–33.

[30] H. Abdi, The eigen decomposition; eigenvalues and eigenvectors. In, *Encylopedia of Measurements and Statistics* (N. J. Salkind, Ed), Sage Publications, Thousand Oaks, California, 2007.

[31] Y. M. A. Hashash, J. I.–C. Yao, and D. C. Wotring, Glyph and hyperstreamline representation of stress and strain tensors and material constitutive response. *Int. J. Num. Anal. Methods Geomech*. 2003, **27**, 604–626.

[32] A. J. Leadbetter and J. E. Spice, The third law entropy and structure of iron pentacarbonyl. *Can. J. Chem.* 1959, **37**, 1923–1929.

[33] W. Cochran, *The Dynamics of Atoms in Crystals.* Arnold, London, 1973.
